# Hepatocyte SAMHD1 Deficiency Attenuates Hepatic Steatosis via Suppression of SREBP Activation in a Mouse Model of Metabolic-Associated Steatotic Liver Disease

**DOI:** 10.7150/ijbs.125688

**Published:** 2026-01-01

**Authors:** Guangfa Yin, Yongqing Liu, Xianhe Teng, Shuqi Sun, Beibei Chen, Xinyu Wang, Tao Yang, Ying Wang, Hanyang Xu, Yu-sheng Chen, Guowei Gan, Yuxian Shen, Juntang Shao

**Affiliations:** 1 School of Pharmacy, Anhui Medical University, 81 Meishan Road, Hefei, 230032, China.; 2 Department of Hepatobiliary and Pancreatic Surgery, The First People's Hospital of Hefei, 390 Huaihe Road, Hefei, 230061, China.; 3 Institute of Health and Medicine, Hefei Comprehensive National Science Center, Northwest Corner of the Intersection of Susong Road and Guanhai Road, Hefei, 230601, China.; 4 Department of General Surgery, The First Affiliated Hospital of Anhui Medical University, 218 Jixi Road, Hefei, 230022, China.

**Keywords:** SAMHD1, metabolic dysfunction-associated steatotic liver disease, hepatic steatosis, SREBP activation, lipid metabolism

## Abstract

Metabolic dysfunction-associated steatotic liver disease (MASLD) is a leading cause of chronic liver disorders and a growing public health concern. Sterile alpha motif and HD domain-containing protein 1 (SAMHD1), a dNTP triphosphohydrolase, is known for its roles in nucleotide metabolism, antiviral defense, and immune regulation, but its function in hepatocytes and contribution to MASLD pathogenesis remain unclear. In this study, we observed that hepatic SAMHD1 expression was markedly increased in MASLD patient samples and diet-induced MASLD mouse models. *In vitro*, mimicking MASLD-associated dyslipidemia with palmitic acid, oleic acid, and cholesterol upregulated SAMHD1 expression, an IFN-γ-induced protein, accompanied by increased IFN-γ receptor 1 expression and STAT1 activation in HepG2 cells. Functional studies using SAMHD1-overexpressing and knockdown hepatic cell lines, as well as hepatocyte-specific AAV-mediated SAMHD1 overexpression *in vivo*, demonstrated that SAMHD1 promoted lipid droplet accumulation. Conversely, hepatocyte-specific SAMHD1 knockout reduced steatosis and liver injury in diet-induced MASLD mouse models. Mechanistically, SAMHD1 enhanced the proteolytic activation of SREBP1 and SREBP2 by upregulating SCAP, S1P, and S2P in a cohesin complex-dependent manner. Collectively, these findings identify hepatocyte SAMHD1 as a promoter of liver steatosis through SREBP activation and highlight it as a potential therapeutic target for MASLD.

## Introduction

Metabolic dysfunction-associated steatotic liver disease (MASLD), also known as nonalcoholic fatty liver disease (NAFLD), is a metabolic stress-induced liver disorder characterized by hepatic steatosis in the absence of excessive alcohol consumption, viral hepatitis, or drug-induced liver injury 1. The global prevalence of MASLD is estimated to be around 30% and continues to rise 2. The disease encompasses a spectrum ranging from isolated hepatic steatosis, which is relatively benign and non-progressive, to metabolic dysfunction-associated steatohepatitis (MASH), a severe form characterized by hepatocellular injury, inflammation, and fibrosis that can progress to cirrhosis and hepatocellular carcinoma (HCC) 3. Currently, drug development strategies for MASH primarily focus on correcting dysregulated glucose and lipid metabolism, as well as anti-fibrotic and anti-inflammatory pathways 4. As of 2025, resmetirom, a liver-targeted selective thyroid hormone receptor-β agonist, and semaglutide, a glucagon-like peptide-1 (GLP-1) receptor agonist, are two FDA-approved treatments for moderate to advanced hepatic fibrosis in MASH 5, 6. Given the significant unmet clinical need, advancing therapeutic options remains a critical priority.

In MASLD, increased *de novo* lipogenesis (DNL) and esterification of fatty acids into triglycerides are key contributors to hepatic steatosis 7. Excess carbohydrate intake, particularly glucose and fructose, combined with elevated insulin levels, stimulates the liver to upregulate key lipogenic genes via sterol regulatory element-binding protein (SREBP, encoded by *SREBF*) and other transcription factors involved in lipid metabolism 8. Once the supply of fatty acids in liver exceeds its metabolic capacity, lipotoxic substances accumulate, leading to endoplasmic reticulum stress, mitochondrial dysfunction, pro-inflammatory cytokine release, and hepatocyte apoptosis, all of which contribute to liver injury 9. Furthermore, dysregulated lipid homeostasis impairs tissue repair pathways, leading to hepatic stellate cell activation and accelerating fibrosis and cirrhosis progression 10. Several emerging therapies aimed at correcting lipid metabolism in MASH are currently in clinical trials, including fibroblast growth factor 21 (FGF21) analogs and pan-peroxisome proliferator-activated receptor (PPAR) agonists 11. Due to the complexity of MASLD pathogenesis and the unmet medical needs in MASH treatment, further investigation into its molecular mechanisms is crucial for identifying new therapeutic targets.

Sterile alpha motif and HD domain-containing protein 1 (SAMHD1) possesses deoxynucleotide triphosphate hydrolase (dNTPase) activity, which plays a role in regulating intracellular dNTP homeostasis 12. By depleting intracellular dNTP pools, SAMHD1 restricts HIV-1 reverse transcription in non-dividing myeloid cells 13. Beyond its antiviral role, SAMHD1 negatively regulates NF-κB signaling pathway 14, facilitates DNA repair 15 and is primarily linked to innate immune disorders, such as Aicardi-Goutières syndrome, as well as resistance to nucleoside analogs-based chemotherapy in cancer 16. Our studies show that SAMHD1 inhibits hepatitis B virus (HBV) replication by restricting the reverse transcription of pregenomic RNA (pgRNA) into relaxed circular DNA (rcDNA) 17, and since HBV is a major HCC risk factor, we further found that nuclear SAMHD1 correlates with favorable prognosis in HCC by interacting with the cohesin complex to enhance chromatid cohesion and stall metaphase progression 18. Despite these findings, the role and molecular mechanisms of SAMHD1 in chronic liver diseases, including MASLD, remain largely unexplored.

In this study, we investigated hepatic SAMHD1 expression in both clinical MASLD samples and diet-induced MASLD mouse models, observing a significant increase. *In vitro*, long-chain fatty acids, cholesterol, and IFN-γ upregulated SAMHD1 in HepG2 cells, and functional assays confirmed its role in promoting lipid droplet accumulation. AAV-mediated overexpression of SAMHD1 exacerbated lipid accumulation through SREBP1/2 activation, while hepatocyte-specific SAMHD1 knockout mice revealed that SAMHD1 deficiency alleviates liver steatosis. Mechanistically, SAMHD1 promotes SREBP proteolytic activation by upregulating SCAP, S1P, and S2P in a cohesin complex-dependent manner.

## Materials and Methods

### Cell culture

HEK293T (GNHu44), HepG2 (TCHu72), and Huh7 (TCHu182) cells were obtained from the Cell Bank of the Chinese Academy of Sciences and cultured in DMEM (C11995500BT, Gibco, Beijing, China) supplemented with 10% fetal bovine serum (FBS; C04001, VivaCell, Shanghai, China), penicillin-streptomycin (C0222, Beyotime, Beijing, China), and 2 mM L-glutamine (C0212, Beyotime). Cells were maintained at 37 °C with 5% CO₂ in tissue culture dishes or multiwell plates (LABSELECT, Hefei, China). Primary mouse hepatocytes were isolated using a two-step collagenase perfusion method 19 and cultured in William's E medium (12551032, Gibco) supplemented with 10% FBS, 1% penicillin-streptomycin, 2 mM L-glutamine, 0.1 µM dexamethasone (ST1254, Beyotime), 10 μg/ml insulin (P3376, Beyotime). SAMHD1-overexpressing (SAMHD1-OE) HepG2 and Huh7 cells were generated by lentiviral transduction with the pLVX-HA-SAMHD1-IRES-Puro expression vector 18. Control cells were transduced with lentivirus encapsulating the empty pLVX-IRES-Puro backbone. Plasmids for SAMHD1 mutants (R451E, T592A, and T592E) were constructed using the pLVX-HA-SAMHD1-IRES-Puro plasmid. Linear DNA sequences encoding SAMHD1 from lysine 332 to the C-terminus (Supplementary Information 1) were synthesized and inserted between the AfeI and BamHI restriction sites by Sangon Biotech (Shanghai, China). SAMHD1-knockdown (SAMHD1-KD) HepG2 and Huh7 cells were generated via lentiviral transduction using the pLKO.1-puro expression vector, which encodes a SAMHD1-specific shRNA. The shRNA sequence consisted of the following sense strand: 5'-GAUUCAUUGUGGCCAUAUA-3' and antisense strand: 5'-UAUAUGGCCACAAUGAAUC-3'.

### Palmitic Acid, oleic Acid, and cholesterol treatment

Cells were seeded in 24-well plates and serum-starved overnight in DMEM containing 0.2% BSA (4240GR025, BioFroxx, Einhausen, Germany). For palmitic acid and oleic acid (PO) treatment, cells were treated for 48 hours with control medium (DMEM supplemented with 10% lipid-depleted FBS, C3840, VivaCell) or with 0.25 mM palmitic acid (PA, P101061, Aladdin, Shanghai, China) and 0.25 mM oleic acid (OA, O431503, Aladdin) in control medium. For cholesterol treatment, cells were treated with control medium or MβCD-cholesterol (C4951, Sigma-Aldrich, Saint Louis, MO, USA) at 125 or 375 µg/mL (equivalent to 5 µg/mL and 15 µg/mL cholesterol, respectively) for 48 hours. After treatment, cells were lysed for western blot analysis or used for RNA extraction for qPCR. The sequences of the primers used are listed in Supplementary Table S2.

### IFN-γ treatment and BODIPY staining

HepG2 cells were seeded in 96-well plates and serum-starved overnight. Cells were then treated for three days with control medium, PO dosing solution, IFN-γ (20 ng/mL, CST80385, Cell Signaling Technology Inc., Massachusetts, USA), or a combination of PO and IFN-γ. Cells were then fixed with 4% paraformaldehyde, stained with 1 µg/mL BODIPY 493/503 (HY-W090090, MCE, Monmouth Junction, New Jersey, USA) and 1 µg/mL DAPI (D9542, Sigma-Aldrich), and imaged using the ImageXpress Micro Confocal System (Molecular Devices, San Jose, California, USA).

### Animal studies

Male C57BL/6J mice (6-8 weeks old, 20-25 g) were obtained from Jiangsu Jicui Yaokang Biotechnology Co., Ltd (Nanjing, China). SAMHD1^flox/flox^ mice, carrying loxP sites flanking exon 3 of the SAMHD1 gene on a C57BL/6J background, were crossed with Alb-Cre mice to generate hepatocyte-specific SAMHD1 knockout (HKO) mice. The efficiency of SAMHD1 knockout in HKO mouse livers and hepatocytes was validated in our previous study 18. SAMHD1^ flox/flox^ mice served as controls (Flox). At 6-8 weeks, corresponding to the onset of diet-induced MASLD, both serum and hepatic levels of triglyceride (TG) and total cholesterol (TC) were comparable between Flox and HKO mice (Supplementary Fig. 1A-D). dNTP content was quantified in primary hepatocytes from Flox and HKO mice using a previously described method 20, showing no significant changes under basal conditions (Supplementary Fig. 1E), This suggests that, despite SAMHD1 being a dNTPase, its effect on intracellular dNTP levels varies by cell type and biological context 21. Mice were housed under specific pathogen-free conditions with a standard 12-hour light/dark cycle and had ad libitum access to distilled water and rodent chow (SWS9102, Xietong Shengwu, Nanjing, China). A mouse MASLD model was established by feeding male mice (6-8 weeks old) a Gubra-Amylin NASH (GAN) diet (40 kcal% fat, 20 kcal% fructose, and 2% cholesterol; D09100310, Research Diets, New Brunswick, USA) for 23 or 30 weeks or a methionine- and choline-deficient, high-fat, high-cholesterol (HCM) diet (45 kcal% fat, 1% cholesterol, 0.1% methionine, and no choline; TP 3622657, TrophicDiet, Nantong, China) for the indicated durations. Mice fed a normal chow diet (SWS9102, Xietong Shengwu) served as controls. Liver tissues were collected at the specified time points for further analyses. Whole blood was obtained via cardiac puncture, and serum was separated by centrifugation at 2000 g for 10 minutes.

AAV2/8 vectors were constructed and purified by OBiO Technology Inc. (Shanghai, China). For hepatocyte-specific SAMHD1 overexpression, a liver-tropic AAV2/8 vector driven by the thyroxine-binding globulin (TBG) promoter (pAAV-TBG-3×FLAG-SAMHD1-P2A-GdGreen-tWPA) was generated. The control vector (pAAV-TBG-GdGreen-tWPA) expressed GdGreen alone to monitor transduction efficiency. Viral particles (2.5 × 10¹¹ vg per mouse) were diluted in sterile saline (50 µL total volume) and administered via tail vein injection. Mice were then maintained under standard housing conditions.

### RNA sequencing

The sequencing was performed by Oebiotech Corporation (Shanghai, China). Total RNA from liver tissue of HKO and Flox mice fed the 23-week GAN diet (Fig. 5K, L) and from isolated primary hepatocytes of HKO and Flox mice (Fig. 6A, B) was extracted using the mirVana miRNA Isolation Kit (Ambion, Austin, TX, USA) according to the manufacturer's protocol. RNA quality was assessed using the Agilent Bioanalyzer 2100 system (Agilent Technologies, Santa Clara, CA, USA), with samples having an RNA integrity number ≥7 being used for further analysis. Libraries were constructed using the TruSeq Stranded mRNA LTSample Prep Kit (Illumina, San Diego, CA, USA) following the manufacturer's instructions. The libraries were then sequenced on the Illumina HiSeq X Ten sequencing platform to generate 150 bp paired-end reads. A p-value < 0.05 and fold change > 2 or < 0.5 were considered the thresholds for significant differential expression. All sequencing reads were exported in FASTQ format. The sequence data have been deposited in the NCBI Sequence Read Archive (SRA) under the accession numbers PRJNA1240341 and PRJNA1240456.

### Biochemical and liver lipid analyses

Serum levels of alanine aminotransferase (ALT) and aspartate aminotransferase (AST) were quantified on a Hitachi automatic analyzer 3100 according to the manufacturer's manuals. Serum TG, TC, and low-density lipoprotein cholesterol (LDL-C) levels were quantified using commercial kits (TG: A110-1-1, TC: A111-1-1, LDL-C: A113-1-1; Jiancheng Bioengineering Institute, Nanjing, China) following the manufacturer's instructions. Liver lipids were isolated using a modified Folch procedure 22. Briefly, Liver samples (50 mg) were homogenized in chloroform/methanol (2:1, v/v), and the lipid-containing chloroform phase was collected and evaporated. Lipids were redissolved in 600 μL ethanol and analyzed using the kits mentioned above.

### Immunohistochemistry and immunofluorescence staining

Paraffin-embedded liver sections (4 µm) were deparaffinized, rehydrated, and subjected to antigen retrieval, followed by blocking of endogenous peroxidase activity. Sections were then incubated overnight at 4°C with primary antibodies, then with secondary antibodies. Detection was performed using DAB substrate (ZLI-9018, Zhongshan Golden Bridge Biotechnology, Beijing, China), with hematoxylin counterstaining. Slides were sealed and scanned using a 3DHISTECH Pannoramic MIDI slide scanner. The list of antibodies is provided in Supplementary Table S3. For immunofluorescence, liver cryosections (8 µm) were fixed in 4% paraformaldehyde, permeabilized with 0.1% Triton X-100, and blocked with goat serum (C0265, Beyotime). After overnight primary antibody incubation at 4°C, slides were incubated with fluorescent secondary antibodies, followed by DAPI staining. Slides were mounted with antifade reagent (P0128S, Beyotime) and imaged on a Zeiss LSM 800 Airyscan confocal microscope (Zeiss, Oberkochen, Germany).

### Oil Red O staining

Liver cryosections or cell samples were fixed in 4% paraformaldehyde, washed with PBS, and stained with Oil Red O working solution (G1260, Solarbio, Beijing, China) for 15 min at room temperature to detect neutral lipid accumulation. Excess stain was removed by washing with 60% isopropanol, and samples were then counterstained with hematoxylin. Images were captured using 3DHISTECH Pannoramic MIDI slide scanner.

### BODIPY and Filipin staining

Liver cryosections were fixed in 4% paraformaldehyde and incubated with BODIPY 493/503 (1 μg/mL) and DAPI (1 μg/mL) for 40 minutes in the dark at room temperature. After PBS washes, slides were mounted with antifade reagent and imaged using confocal microscopy. For Filipin staining, cryosections were incubated with 0.25 mg/mL Filipin (HY-N6716, MCE) in PBS for 1 hour at room temperature in the dark, followed by mounting and confocal imaging.

### TUNEL staining

Liver sections were deparaffinized, rehydrated, and treated with proteinase K (20 μg/mL, ST533, Beyotime) at 37°C for 15-30 minutes. After washing with PBS, TUNEL reaction mixture (C1088, Beyotime) was applied and incubated at 37°C for 60 minutes in the dark. Nuclei were counterstained with Hoechst 33342 (BL803A, Biosharp, Beijing, China) for 5 minutes, mounted with antifade reagent, sealed, and imaged using a fluorescence microscope.

### Quantitative real time polymerase chain reaction (qPCR)

Total RNA was extracted using TRIzol reagent (15596018, Thermo Fisher) and reverse transcription was performed with the PrimeScript RT Reagent Kit (A230, Genstar, Beijing, China) according to the manufacturer's instructions. qPCR was conducted using the SYBR qPCR Mix Kit (A301, Genstar). Primer sequences are provided in Supplementary Table S2.

### Dual luciferase reporter assay

The 5' upstream regulatory regions of SREBP1 (-2,000 bp to +169 bp) and SREBP2 (-2,000 bp to +166 bp) were inserted into the pGL4.17[luc2/Neo] vector containing the firefly luciferase gene, respectively (schematic in Supplementary Fig. 1W, X). The phRL-TK vector with Renilla luciferase was used as an internal control in the dual luciferase assay. 800 ng of pcDNA3.1 SAMHD1 plasmid, 800 ng of pGL4.17[luc2/Neo]-SREBP1 or SREBP2 promoter, and 20 ng of phRL-TK were co-transfected into HEK293T cells using Lipofectamine 2000, with empty pcDNA3.1 as a negative control and cells treated with 100 nM insulin (P3376, Beyotime) as a positive control for activating the SREBP promoter. 48 hours after transfection, luciferase activity was detected using the Dual-Glo luciferase assay kit (E2920, Promega, Madison, USA), and relative luciferase activity was calculated based on the ratio of firefly luciferase activity to Renilla luciferase activity.

### CHX chase assay

HepG2 cells were treated with cycloheximide (CHX, 50 µg/mL, MS-0035, MKbio) to inhibit protein synthesis. At time points of 0, 1, 3, 6, 9, and 12 hours, cells were lysed, and total protein was extracted for western blot analysis.

### Study approval

Normal control liver specimens and liver specimens from suspected adult MASLD patients were obtained during gallbladder excision surgery due to gallstones. Individuals with excessive alcohol intake, or other liver diseases (e.g., hepatitis B, hepatitis C) were excluded. Written informed consent was obtained from each participant for sample collection. Ethics approval (20210515) was granted by Anhui Medical University (Hefei, China) for the use of clinical samples in research. Animal studies were approved by the Animal Care and Use Committee of Anhui Medical University (LLSC20250602).

### Statistical analysis

Statistical analyses were conducted using GraphPad Prism (version 10). Data normality was assessed with QQ plots, and variance homogeneity was tested using the F-test. Results are presented as mean ± standard deviation (SD). Comparisons between two groups were performed using two-tailed Student's t-tests—unpaired t-tests for equal variances and Welch's t-tests when variances differed. Details on sample sizes and significance levels are provided in the figure legends.

## Results

### Hepatic SAMHD1 is upregulated in MASLD patients and correlates with steatosis severity in diet-induced MASLD mouse models

**Figure 1:**
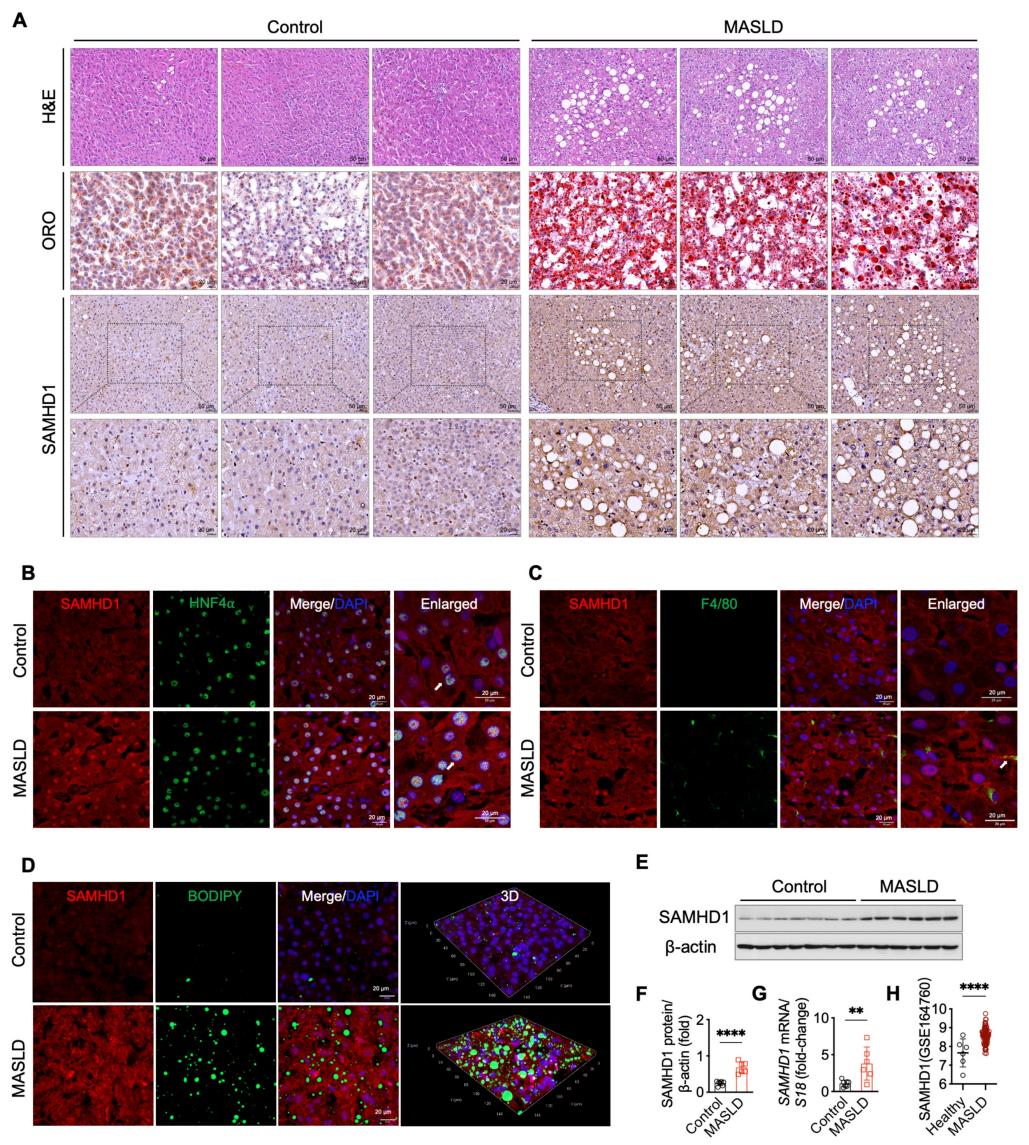
** SAMHD1 expression was upregulated in fatty liver in MASLD patients.** (A) Representative images of H&E staining, Oil Red O (ORO) staining, and immunohistochemical (IHC) staining for SAMHD1 in liver sections from non-MASLD (control) and MASLD individuals. Scale bars: 50 µm for H&E staining, 20 µm for ORO staining. Scale bars for SAMHD1 IHC staining: 50 µm (upper panel) and 20 µm (lower panel). (B) Immunofluorescence staining of liver sections for SAMHD1 and HNF4α. Scale bars: 20 µm. (C) Immunofluorescence staining of liver sections for SAMHD1 and F4/80. Arrows indicate representative SAMHD1 expression in HNF4α⁺ or F4/80⁺ cells. Image of negative control staining using isotype control antibodies are provided in Supplementary Fig.1F. Scale bars: 20 µm. (D) Immunofluorescence staining of liver sections for SAMHD1, combined with BODIPY staining to visualize lipid droplets. Representative images and three-dimensional reconstruction from Z-stack images are shown. Scale bars: 20 µm. (E) Representative western blot analysis of SAMHD1 expression in liver samples from control and MASLD individuals. (F) Quantification of SAMHD1 protein expression based on western blot analysis. n = 7 for the control group, n = 6 for the MASLD group. (G) Relative mRNA expression levels of SAMHD1 in liver tissues from control and MASLD individuals. (H) Scatter plots showing SAMHD1 expression levels in the GEO dataset GSE164760. The y-axis represents log2-transformed RMA-normalized expression values of SAMHD1, derived using the RMA algorithm and quantile normalization. RMA: Robust Multi-array Average. Control: n = 6; MASLD: n = 74. Data are presented as means ± SD. p < 0.01 (**), p < 0.001 (***). Two-tailed Student's t-test was used for two-group comparisons in (F-H).

To investigate the role of SAMHD1 in MASLD, we evaluated its expression in liver samples from MASLD patients and non-MASLD controls, with baseline characteristics summarized in Supplementary Table S1. Histological analysis, including H&E, Oil Red O, and BODIPY staining, confirmed the presence of pronounced hepatic steatosis in MASLD patients (Fig. 1A, D). Single-cell RNA sequencing data revealed that SAMHD1 is predominantly expressed in Kupffer cells, monocytes, lymphocytes, and hepatocytes within the liver (data available at https://www.proteinatlas.org/ENSG00000101347-SAMHD1/single+cell). In line with these results, IHC analysis showed low SAMHD1 expression in both parenchymal and non-parenchymal cells of control livers, whereas MASLD livers exhibited significantly increased SAMHD1 staining (Fig. 1A). Double immunofluorescence staining confirmed elevated SAMHD1 expression in hepatocytes (Fig. 1B). Although SAMHD1 is generally considered a nuclear protein due to its N-terminal nuclear localization sequence (¹¹KRPR¹⁴), it has been detected in both the nucleus and cytoplasm of various cell types, including CD4⁺ T cells and macrophages 23. Consistent with these observations, IHC and double immunofluorescence staining revealed SAMHD1 localization in both the nucleus and cytoplasm of hepatocytes, as previously observed in hepatocytes from adjacent non-tumor tissues of HCC patient and control mouse livers 18. Moreover, the number of F4/80⁺ macrophages was increased in MASLD livers, with prominent SAMHD1 expression observed in these cells (Fig. 1C). Elevated hepatic SAMHD1 expression in MASLD livers was also confirmed by western blot, qPCR, and immunostaining (Fig. 1D-G). Analysis of the publicly available Gene Expression Omnibus dataset (GSE164760) also revealed significantly higher SAMHD1 mRNA levels in MASLD liver tissues compared to controls (Fig. 1H).

**Figure 2:**
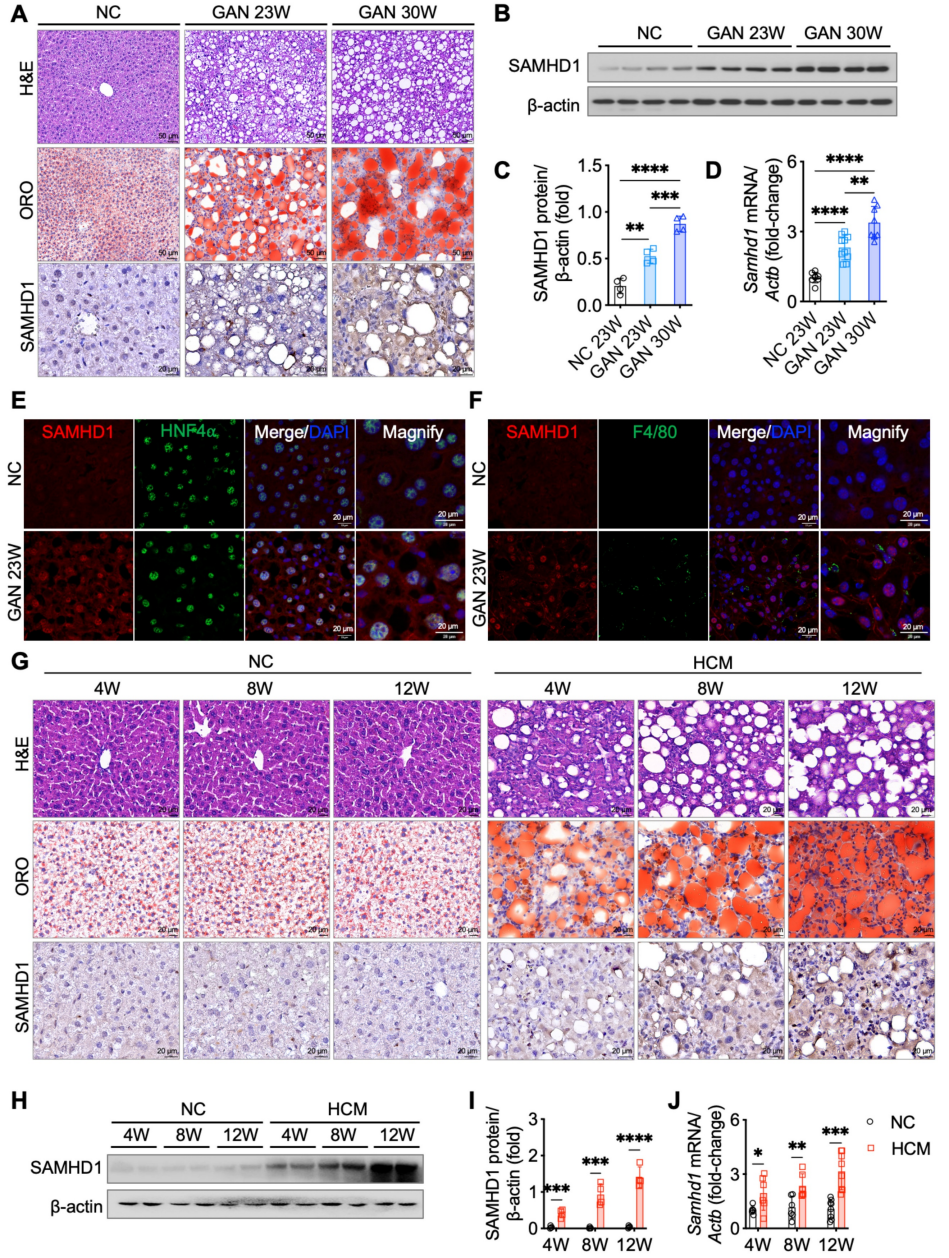
** Hepatic SAMHD1 levels were upregulated in diet-induced MASLD mouse models and progressively increased with steatosis severity.** (A) Representative H&E, ORO, and IHC staining for SAMHD1 in liver sections from mice fed a normal chow (NC) diet or a GAN diet for 23 or 30 weeks. Scale bars: 50 µm (H&E, ORO), 20 µm (IHC). (B, C) Representative western blot and corresponding quantification of SAMHD1 protein levels in liver tissues from mice in indicated groups. n = 4/group. (D) Relative SAMHD1 mRNA levels in liver tissues from the indicated groups; n = 8 (NC), 10 (GAN 23W), and 8 (GAN 30W). (E) Immunofluorescence staining of SAMHD1 and HNF4α in liver sections. Scale bar: 20 µm. (F) Immunofluorescence staining of SAMHD1 and F4/80 in liver sections. Scale bar: 20 µm. (G) Representative H&E, ORO, and SAMHD1 IHC staining of liver sections from mice fed NC or HCM diets for the indicated durations. Scale bar: 20 µm. (H, I) Representative western blots and quantification of SAMHD1 protein levels in liver samples from the indicated groups (n = 4 per group). (J) Relative SAMHD1 mRNA levels in the same groups (n = 6 for 4- and 8-week diets; n = 8 for 12-week diet). Data are presented as means ± SD. p < 0.05 (*), p < 0.01 (**), p < 0.001 (***), and p < 0.0001 (****). Two-tailed Student's t-test was used for two-group comparisons in (C), (D), (I), and (J).

To explore the relationship between hepatic SAMHD1 expression and MASLD progression, we conducted longitudinal studies using two diet-induced MASLD mouse models. In the GAN diet model, which is designed to induce MASLD with a high-fat, high-fructose, and high-cholesterol diet, liver steatosis developed by week 23, as shown by H&E staining revealing prominent fat vacuoles (Fig. 2A). Oil Red O staining also demonstrated a gradual increase in lipid droplet accumulation, indicating worsening steatosis. Western blot, qPCR, and IHC analyses revealed elevated hepatic SAMHD1 expression in the GAN diet group compared to normal chow (NC)-fed controls, with expression levels progressively rising alongside the development of hepatic steatosis (Fig. 2A-D). Immunofluorescence staining confirmed increased SAMHD1 expression in both hepatocytes and macrophages (Fig. 2E, F). In the second model, mice on HCM diet developed liver steatosis by week 4, as evidenced by H&E staining showing significant fat vacuoles and Oil Red O staining confirming progressive lipid droplet accumulation (Fig. 2G). Western blot, qPCR, and IHC analyses revealed upregulation of SAMHD1 expression in the HCM diet group compared to NC-fed controls, with levels rising in parallel with the development of steatosis (Fig. 2G-J). Additionally, analysis of the GSE167523 dataset, comprising liver transcriptomic data from MASLD patients with simple steatosis (n = 51) and MASH (n = 47), revealed significantly higher SAMHD1 expression in MASH livers, further supporting the association between SAMHD1 upregulation and disease severity (Supplementary Fig. 1G).

### Fatty acids, cholesterol, and IFN-γ induce SAMHD1 expression in HepG2 cells

**Figure 3:**
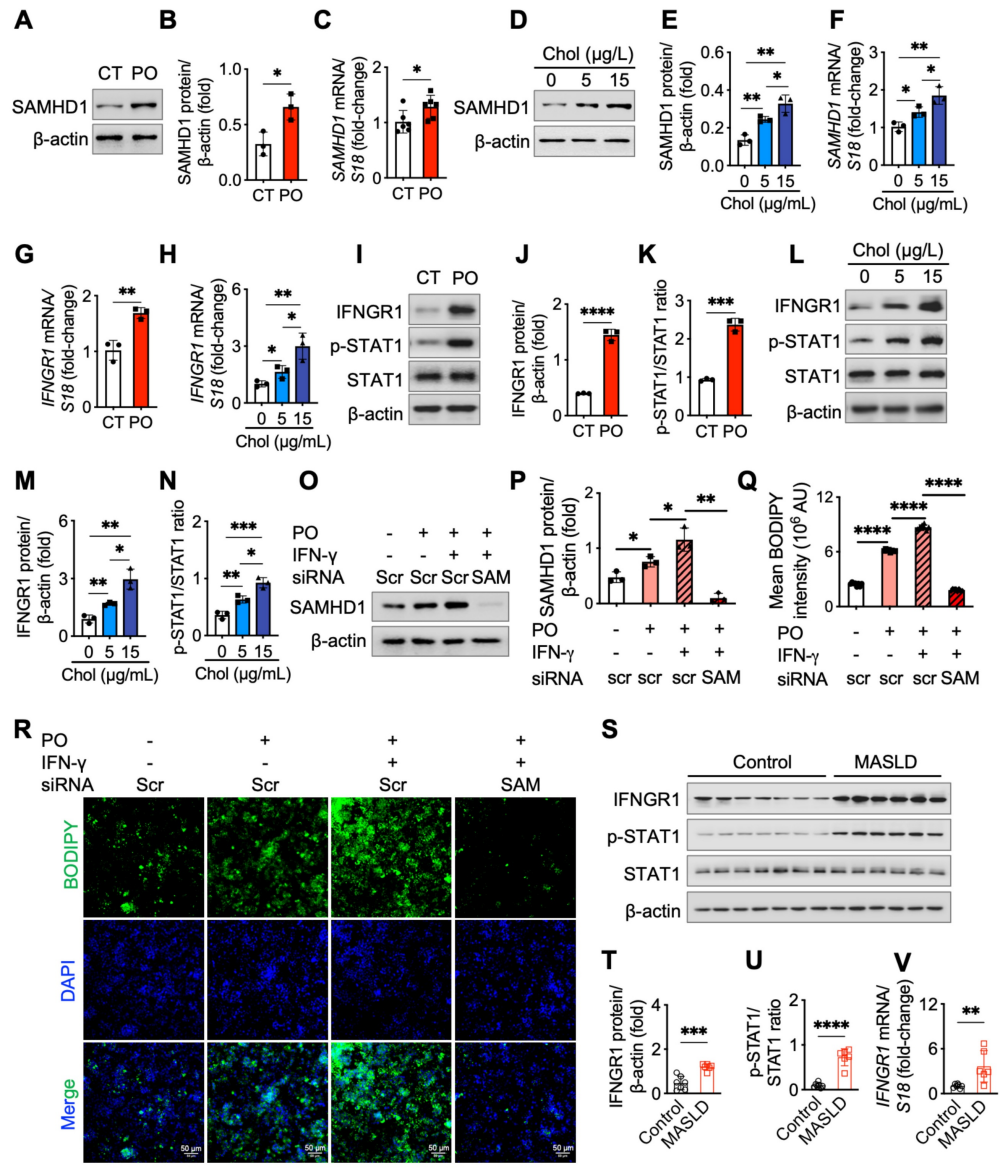
** Palmitic acid and oleic Acid, cholesterol, and IFN-γ upregulated SAMHD1 expression.** (A, B) Representative western blot and quantification of SAMHD1 protein levels (n = 3/group), and (C) SAMHD1 mRNA expression in HepG2 cells cultured in control medium (CT, DMEM + 10% delipidated FBS) or lipid-rich medium containing 0.25 mM palmitic acid (PA) and 0.25 mM oleic acid (OA) (PO) (n = 6/group). (D, E, F) Western blot and quantification of SAMHD1 protein levels and mRNA expression in HepG2 cells cultured in delipidated FBS DMEM or treated with cholesterol (5 µg/mL or 15 µg/mL) (n = 3/group). (G, H) Relative IFNGR1 mRNA levels in HepG2 cells treated with PO or cholesterol (n = 3/group). (I, J, K) Western blot and quantification of IFNGR1, phosphorylated STAT1, and total STAT1 in HepG2 cells treated with PO (n = 3/group). (L, M, N) Western blot and quantification of IFNGR1, phosphorylated STAT1, and total STAT1 in HepG2 cells treated with cholesterol (n = 3/group). (O, P) Representative western blot and quantification of SAMHD1 protein levels in HepG2 cells transfected with SAMHD1 or control siRNA, with or without PO or IFN-γ treatment (n = 3/group). (Q, R) Representative BODIPY staining of HepG2 cells treated as described in (P). Scale bar: 50 µm. Quantification of BODIPY-positive area (n = 8/group). (S, T, U) Western blot analysis and quantification of IFNGR1, phosphorylated STAT1, and total STAT1 in liver samples from control and MASLD individuals. (V) Relative IFNGR1 mRNA levels in liver samples from control and MASLD individuals. Data are presented as means ± SD. Statistical significance is indicated as follows: *p < 0.05, **p < 0.01, ***p < 0.001, and ****p < 0.0001. Two-tailed Student's t-test was used for two-group comparisons in (B), (C), (E-H), (J), (K), (M), (N), (P), (Q), and (T-V).

To investigate factors that upregulate SAMHD1 expression, we first examined its levels in HepG2 cells cultured in a lipid-rich environment, mimicking the conditions of MASLD by supplementing the control medium with long-chain fatty acids or cholesterol. Western blot and qPCR analyses revealed that treatment with palmitic acid and oleic acid (PO) significantly increased both SAMHD1 protein and mRNA levels (Fig. 3A-C), a pattern that was similarly observed in primary mouse hepatocytes (Supplementary Fig. 1H, I). Moreover, cholesterol treatment also resulted in a dose-dependent upregulation of SAMHD1 expression (Fig. 3D-F). Given that SAMHD1 is a well-known interferon-stimulated gene (ISG) in monocytes, macrophages, and dendritic cells, we next investigated whether IFN-γ could similarly upregulate SAMHD1 expression in HepG2 and Huh7 cells. Indeed, IFN-γ treatment significantly enhanced SAMHD1 protein and mRNA levels (Supplementary Fig. 1J, K).

We then investigated whether the increase in SAMHD1 expression induced by PO and cholesterol was associated with potentiation of the IFN-γ signaling pathway. qPCR and western blot analyses showed that both PO and cholesterol treatments upregulated Interferon Gamma Receptor 1 (IFNGR1) expression in HepG2 cells (Fig. 3G, H), with no change in IFNGR2 levels (Supplementary Fig. 1L, M). This was accompanied by greater phosphorylation of STAT1 (Fig. 3I-N), indicating lipid supplementation enhanced IFN-γ signaling activity. Consistently, primary mouse hepatocytes treated with PO also showed elevated IFNGR1 expression and enhanced IFN-γ signaling activity, as indicated by an increased phospho-STAT1 ratio (Supplementary Fig. 1N, O). To further validate this, we added exogenous IFN-γ to the medium and observed an additive effect on SAMHD1 upregulation in response to PO treatment (Fig. 3O, P). Additionally, BODIPY staining demonstrated that IFN-γ treatment enhanced PO-induced lipid droplet accumulation in HepG2 cells (Fig. 3Q, R). This effect was reversed upon SAMHD1 knockdown, indicating that SAMHD1 is crucial for IFN-γ-mediated lipid droplet accumulation under lipid-rich conditions. Moreover, potentiation of the IFN-γ signaling pathway, indicated by increased IFNGR1 expression and a higher phospho-STAT1 ratio, was observed in liver lysates from both MASLD patient samples (Fig. 3S-V) and the GAN-diet-induced mouse MASLD model (Supplementary Fig. 1P-S).

### Hepatocyte SAMHD1 promotes lipid accumulation *in vitro* and *in vivo*

**Figure 4:**
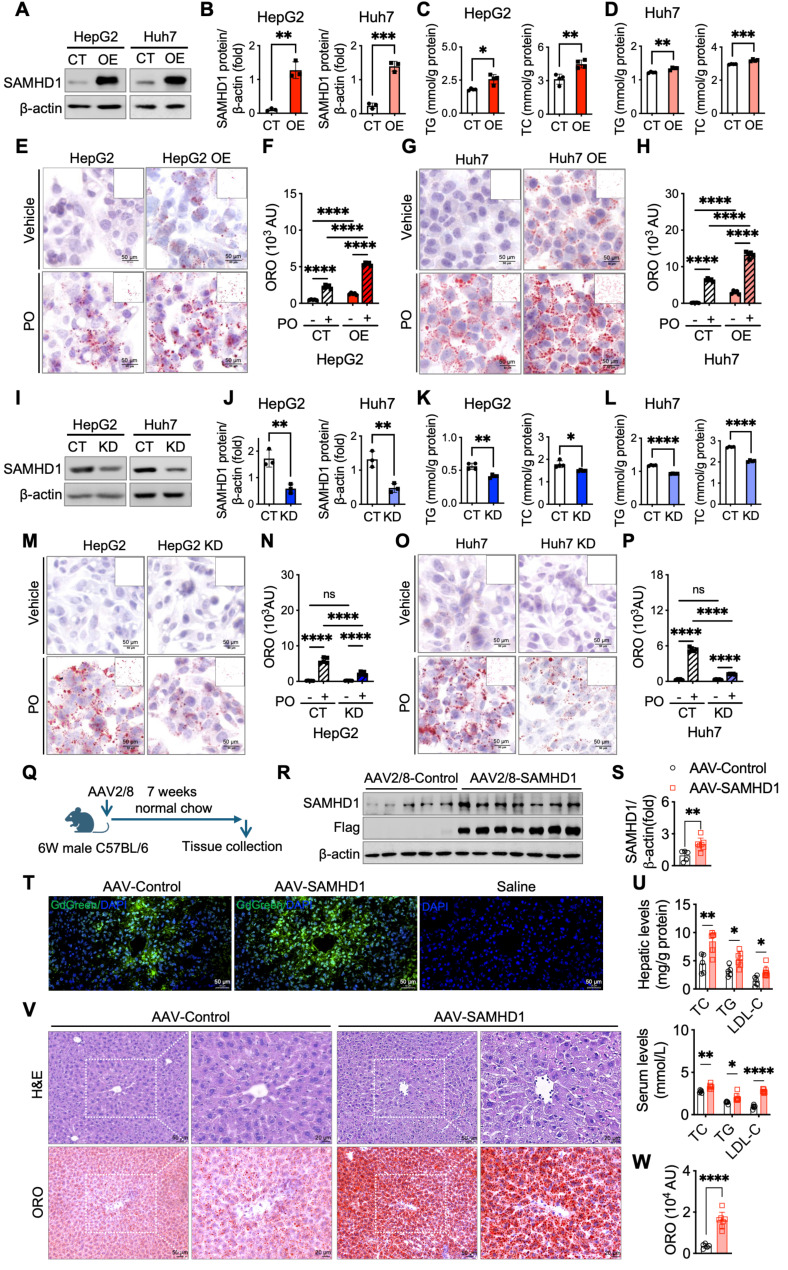
** Hepatocyte SAMHD1 promoted lipid accumulation *in vitro* and *in vivo*.** (A) Representative western blot images showing SAMHD1 protein levels in SAMHD1-overexpressing (SAMHD1-OE) HepG2 and Huh7 cells, compared to control (CT) cells. (B) Quantification of SAMHD1 protein expression normalized to β-actin (n = 3/group). (C, D) Quantification of intracellular triglycerides (TG) and total cholesterol (TC) in SAMHD1-OE and CT HepG2 (C) and Huh7 (D) cells (n = 4/group). (E, G) Representative ORO staining images of CT or SAMHD1-OE HepG2 (E) and Huh7 (G) cells, with or without PO treatment. Scale bars: 50 µm. Insets show ImageJ-processed images highlighting ORO-stained lipid accumulation (red). (F, H) Quantification of ORO staining areas using ImageJ software (n = 5/group). (I) Representative western blot images showing SAMHD1 protein levels in SAMHD1-knockdown (SAMHD1-KD) HepG2 and Huh7 cells, compared to control (CT) cells. (J) Quantification of SAMHD1 protein expression normalized to β-actin (n = 3/group). (K, L) Quantification of intracellular TG and TC in SAMHD1-KD and CT HepG2 (K) and Huh7 (L) cells (n = 4/group). (M, O) Representative ORO staining images of CT or SAMHD1-KD HepG2 (M) and Huh7 (O) cells, with or without PO treatment. Scale bars: 50 µm. (N, P) Quantification of ORO staining areas using ImageJ software (n = 5/group). (Q) Schematic representation of hepatocyte SAMHD1 overexpression via tail vein injection of TBG-SAMHD1 AAV2/8. (R, S) Representative western blot images showing SAMHD1 and Flag-tag levels in liver lysates from AAV-Control and AAV-SAMHD1 mice, with corresponding quantification normalized to β-actin (n = 5 and n = 7, respectively). (T) Representative fluorescence images showing GFP expression in liver sections from AAV-Control, AAV-SAMHD1, or saline-injected mice. (U) Hepatic and serum TC, TG, and LDL-C levels in AAV-Control and AAV-SAMHD1 mice. (V) Representative H&E and ORO staining images of liver sections from the indicated groups. Scale bars: 50 µm and 20 µm. (W) Quantification of ORO staining areas using ImageJ. AU: arbitrary units. Data are presented as means ± SD. p < 0.05 (*), p < 0.01 (**), p < 0.001 (***), and p < 0.0001 (****). Two-tailed Student's t-test was used for two-group comparisons in (B-D), (F), (H), (J-L), (N), (P), (S), (U) and (W).

To directly assess the role of SAMHD1 in lipid accumulation, we generated HepG2 and Huh7 cells with SAMHD1 overexpression (OE) or knockdown (KD) (Fig. 4A, B, I, J). In cells cultured in DMEM with 10% FBS, SAMHD1 overexpression increased TG and TC levels, whereas SAMHD1 knockdown reduced them (Fig. 4C, D, K, L). Following overnight serum starvation, PO-induced lipid droplet accumulation was evaluated. Oil Red O staining showed a marked increase in lipid droplets in SAMHD1-OE cells under both basal and PO-treated conditions (Fig. 4E-H), while SAMHD1 knockdown decreased PO-induced lipid droplet formation (Fig. 4M-P), indicating that SAMHD1 enhances lipid accumulation *in vitro*. Consistently, SAMHD1 knockout in primary mouse hepatocytes treated with PO also display less lipid droplet formation (Supplementary Fig. 1T). To determine whether hepatocyte SAMHD1 promotes lipid accumulation *in vivo*, we administered a liver-targeted AAV2/8 vector carrying a TBG promoter-driven SAMHD1 expression cassette. Delivery was confirmed by GdGreen fluorescence in mice injected with either control GdGreen or SAMHD1-Flag-GdGreen constructs (Fig. 4T), and overexpression was validated by immunoblotting for SAMHD1 and Flag (Fig. 4R, S). Mice with hepatocyte-specific SAMHD1 overexpression exhibited significantly elevated serum and hepatic TG, TC, and LDL-C levels (Fig. 4U), along with enhanced hepatic lipid droplet accumulation as shown by Oil Red O staining (Fig. 4V, W).

### Hepatocyte-specific SAMHD1 deficiency alleviates liver steatosis in GAN diet-induced MASLD mouse model

**Figure 5:**
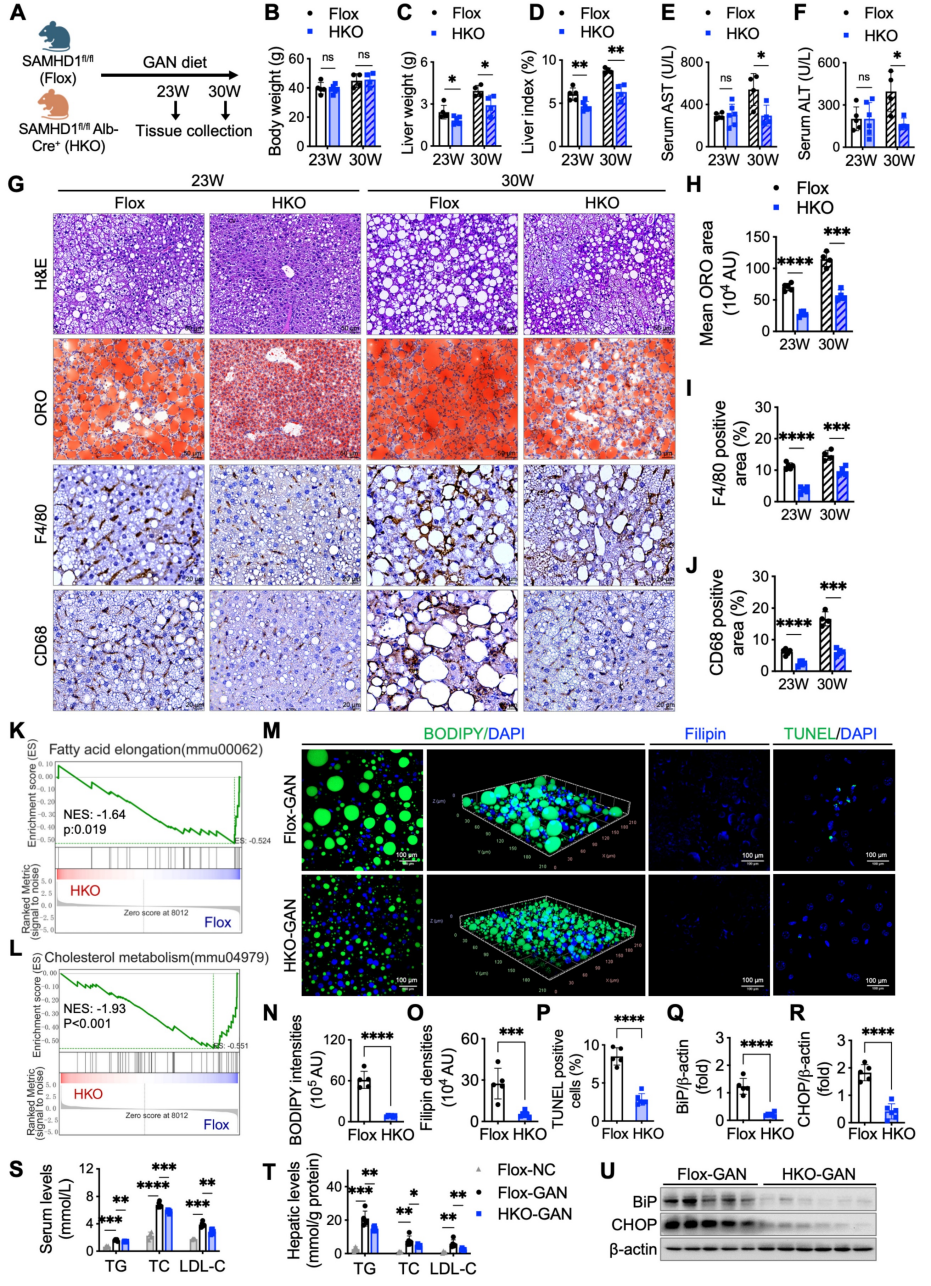
** Hepatocyte-specific SAMHD1 deficiency alleviated liver steatosis in GAN diet-induced MASLD mouse model.** (A) Schematic representation of the GAN diet-induced MASLD model in Flox and HKO mice. (B-D) Comparison of (B) body weight, (C) liver weight, and (D) liver index (liver weight/body weight) in Flox and HKO mice fed a GAN diet for the indicated durations. Sample sizes for each group: Flox-23 weeks (n=5), HKO-23 weeks (n=6), Flox-30 weeks (n=4), HKO-30 weeks (n=4). (E, F) Serum levels of aspartate aminotransferase (AST) and alanine aminotransferase (ALT) in Flox and HKO mice on the GAN diet over time. (G) Representative images of liver sections stained with H&E, ORO, and IHC for F4/80 and CD68. Scale bars: 50 µm (H&E and ORO), 20 µm (IHC). (H-J) Quantification of ORO staining area (H), F4/80-positive cell area (I), and CD68-positive cell area (J). (K, L) Bulk RNA sequencing (RNA-seq) analysis comparing Flox and HKO mice on a GAN diet for 23 weeks, with gene set enrichment analysis (GSEA) performed using indicated hallmark gene sets. (M) Representative images of BODIPY staining and 3D reconstruction of Z-stack images, along with Filipin and TUNEL staining. Scale bars: 100 µm. (N-P) Quantification of BODIPY-positive lipid accumulation (N), Filipin-positive cholesterol deposition (O), and TUNEL-positive apoptotic cells (P), calculated as a percentage from high-power fields (400X). (Q, R, U) Representative western blot images showing BiP and CHOP protein levels in Flox and HKO mice after 23 weeks on a GAN diet, with corresponding quantification normalized to β-actin. (S, T) Serum (S) and hepatic (T) triglyceride (TG), total cholesterol (TC), and low-density lipoprotein cholesterol (LDL-C) levels in Flox and HKO mice fed either a normal chow or GAN diet for 23 weeks. Data are presented as means ± SD. p < 0.05 (*), p < 0.01 (**), p < 0.001 (***), and p < 0.0001 (****). Two-tailed Student's t-test was used for two-group comparisons in (B-F), (H-J), and (N-T).

To investigate the role of hepatocyte SAMHD1 in MASLD progression, we generated hepatocyte-specific SAMHD1-knockout (HKO) mice 18. Compared to their SAMHD1^flox/flox^ (Flox) littermate controls, HKO mice exhibited reduced liver weight and liver index at both 23 and 30 weeks on the GAN diet (Fig. 5A-D), while body weight and food intake remained similar between the groups (Supplementary Fig. 1U, V). At 30 weeks, Flox mice showed significantly elevated serum alanine ALT and AST levels compared to their 23-week counterparts, indicating progressive liver injury. In contrast, HKO mice exhibited significantly lower ALT and AST levels than Flox controls at 30 weeks, but no difference was observed between HKO and Flox mice at 23 weeks, suggesting that hepatocyte SAMHD1 deficiency mitigates GAN diet-induced liver damage as the diet progresses (Fig. 5E, F). Histological analysis further supported these findings. Oil Red O staining and IHC for F4/80 and CD68 revealed reduced liver steatosis and decreased macrophage activation and infiltration in HKO mice at both time points (Fig. 5G-J), emphasizing the protective role of SAMHD1 deficiency against hepatic lipid accumulation and inflammation.

To investigate the broader molecular changes underlying liver injury and lipid metabolism in HKO mice, gene set enrichment analysis (GSEA) of RNA sequencing data from liver tissues of HKO and Flox mice after 23 weeks on the GAN diet revealed significant differential regulation of genes involved in lipid metabolism, including fatty acid synthesis and cholesterol metabolism (Fig. 5K, L). BODIPY and Filipin staining confirmed reduced lipid and cholesterol accumulation in HKO livers at 23 weeks (Fig. 5M-O), which was consistent with lower TG, TC and LDL-C levels in both serum and liver tissues (Fig. 5S, T). Furthermore, TUNEL staining indicated fewer apoptotic cells in HKO livers at 23 weeks (Fig. 5M, P), along with reduced expression of BiP and CHOP, markers of endoplasmic reticulum (ER) stress (Fig. 5Q, R, U). These data, along with the findings at 30 weeks, suggest that hepatocyte-specific SAMHD1 deficiency alleviates liver steatosis, reduces lipid and cholesterol accumulation, and mitigates liver injury in the GAN diet-induced MASLD mouse model.

### Hepatocyte SAMHD1 enhances SREBP1 and SREBP2 signaling pathways

**Figure 6:**
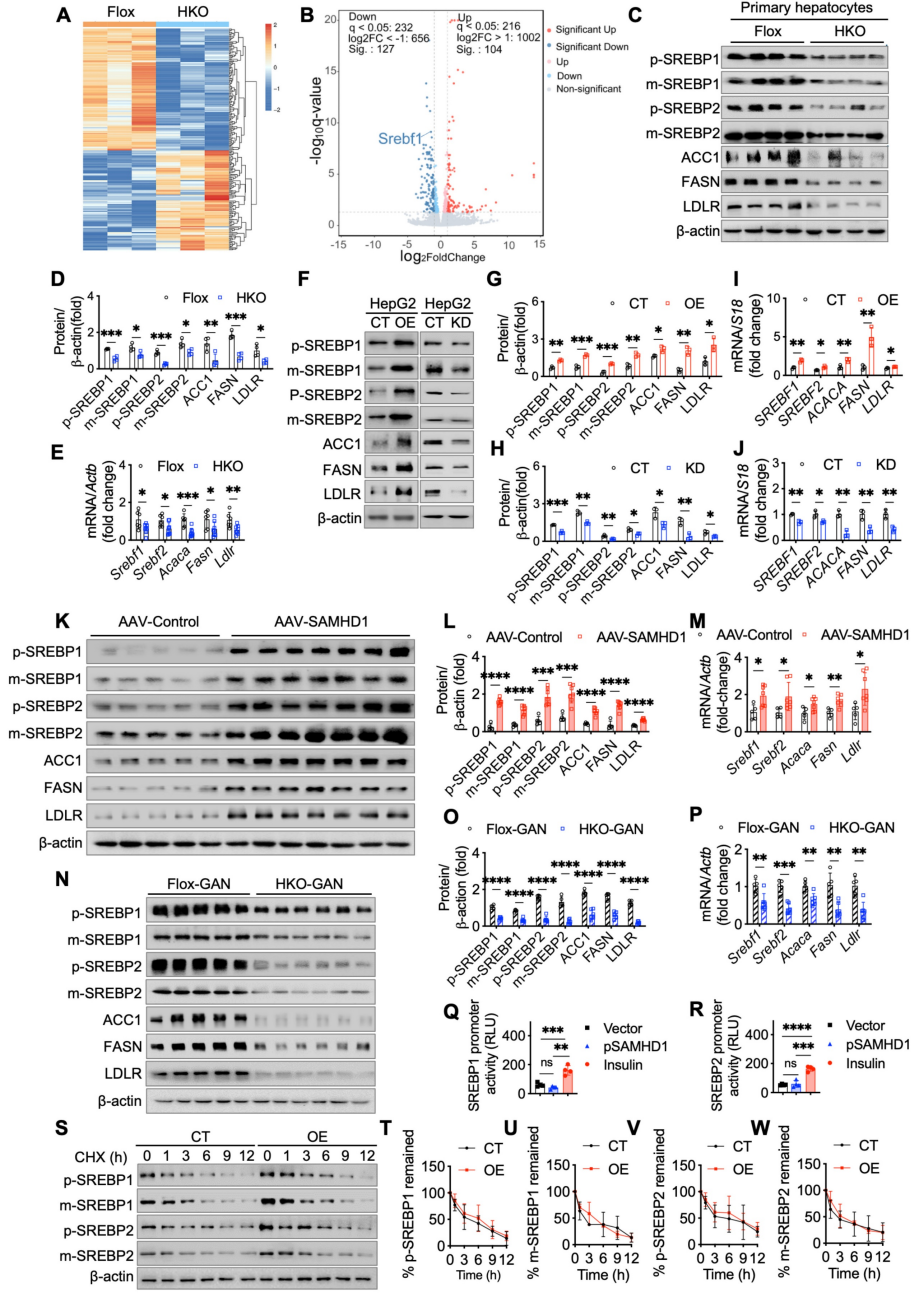
** Hepatocyte SAMHD1 enhanced SREBP1 and SREBP2 signaling pathways.** (A) Heatmap showing hierarchical clustering of differentially expressed genes between primary hepatocytes from HKO and Flox mice. (B) Volcano plot highlighting differentially expressed genes, including *Srebf1*, between HKO and Flox mice. (C, D) Representative western blot showing precursor (p-) and mature (m-) forms of SREBP1, SREBP2, and their downstream target proteins in primary hepatocytes from Flox and HKO mice, with quantification normalized to β-actin (n=4/group). (E) Relative mRNA expression levels of SREBP1, SREBP2, and their downstream targets in primary hepatocytes from Flox and HKO mice (n=4/group). (F-H) Representative western blot images showing SREBP1, SREBP2, and their downstream target protein levels in SAMHD1-overexpressing (OE) and control (CT) HepG2 cells, as well as in SAMHD1-knockdown (KD) and CT HepG2 cells, with corresponding quantification normalized to β-actin (n=3/group). (I, J) Relative mRNA expression levels of SREBP1, SREBP2, and their downstream targets in SAMHD1-OE and CT HepG2 cells, as well as in SAMHD1-KD and CT HepG2 cells (n=3/group). (K, L, M) Representative western blot images and quantification of SREBP1, SREBP2, and downstream target protein levels, along with relative mRNA levels, in liver lysates from AAV-Control and AAV-SAMHD1 mice. (N, O, P) Representative western blot images and quantification of SREBP1, SREBP2, and their downstream target protein levels, along with relative mRNA expression, in liver lysates from Flox and HKO mice fed a GAN diet for 23 weeks. (Q, R) Luciferase reporter activities for SREBP1 (Q) or SREBP2 (R) promoters cloned in the pGL4.17[luc2/Neo] vector, transfected into HEK293T cells with Renilla and either SAMHD1-expressing or vector control pcDNA3.1. Insulin (100 nM) was used as a positive control (n=4/group). (S-W) Representative western blot images of a cycloheximide (CHX) chase assay in SAMHD1-OE and CT HepG2 cells, with corresponding quantification normalized to β-actin at time zero. The intensity values at 0 h (lane 1 for CT cells; lane 7 for OE cells) were set to 100%, and the intensities of all other bands were normalized accordingly. Plots represent the average values from three independent experiments. Data are presented as means ± SD. p < 0.05 (*), p < 0.01 (**), p < 0.001 (***), and p < 0.0001 (****). Two-tailed Student's t-test was used for two-group comparisons in (D), (E), (G-J), (L), (O-R) and (T-W).

To explore the specific molecular mechanisms by which SAMHD1 regulates lipid metabolism, we performed RNA sequencing on primary hepatocytes isolated from HKO and Flox mouse livers to identify differentially expressed genes (Fig. 6A). Our analysis identified *Srebf1*, a key regulator of *de novo* fatty acid synthesis, as significantly downregulated in HKO hepatocytes (Fig. 6B). Western blot and qPCR analyses confirmed these findings, showing reduced levels of both the full-length precursor (inactive form) and cleaved (active form) of SREBP1 in HKO hepatocytes (Fig. 6C, D). Additionally, key SREBP1 downstream targets, including acetyl-CoA carboxylase 1 (ACC1) and fatty acid synthase (FASN)—crucial enzymes in lipid synthesis—were also significantly reduced in HKO hepatocytes. Interestingly, SREBP2, a core transcription factor involved in cholesterol metabolism, was similarly downregulated in HKO hepatocytes, with decreased levels of both its active cleaved form and precursor (Fig. 6C, D). This was accompanied by reduced expression of its downstream target, the low-density lipoprotein receptor (LDLR), indicating a broader impact on lipid homeostasis. qPCR further validated the downregulation of SREBP1/2 and their respective targets in HKO hepatocytes (Fig. 6E). Consistent with these findings, SAMHD1-overexpressing HepG2 cells showed elevated levels of both precursor and cleaved forms of SREBP1 and SREBP2, along with increased expression of their downstream targets ACC1, FASN, and LDLR (Fig. 6F, G, I). Conversely, SAMHD1 knockdown in HepG2 cells led to decreased expression of both SREBP1 and SREBP2, as well as their downstream targets (Fig. 6F, H, J).

*In vivo*, hepatocyte-specific overexpression of SAMHD1 showed elevated hepatic SREBP activation, with increased levels of both precursor and cleaved forms of SREBP1 and SREBP2, as well as upregulation of their downstream targets (Fig. 6K-M). Finally, we assessed SREBP1/2 expression in HKO and Flox mice after 23 weeks on the GAN diet. Western blot and qPCR analyses confirmed that SREBP1/2 and their downstream targets were significantly downregulated in HKO mouse livers compared to Flox controls (Fig. 6N-P), linking SAMHD1 in hepatocytes to lipid metabolism in the context of MASLD.

### SAMHD1 enhances SREBP1 and SREBP2 activation by promoting their proteolytic cleavage through upregulation of SCAP, S1P, and S2P

We next investigated the mechanisms by which SAMHD1 enhances SREBP1 and SREBP2 signaling. To determine whether SAMHD1 directly regulates their transcription, dual-luciferase reporter assays were performed. Overexpression of SAMHD1 did not increase the activity of the SREBP1 or SREBP2 promoters (Fig. 6Q, R). To assess effects on protein stability, CHX chase assays were conducted. No significant differences in SREBP1/2 turnover were observed between control and SAMHD1-overexpressing HepG2 cells, indicating that SAMHD1 does not affect protein stability (Fig. 6S-W).

**Figure 7:**
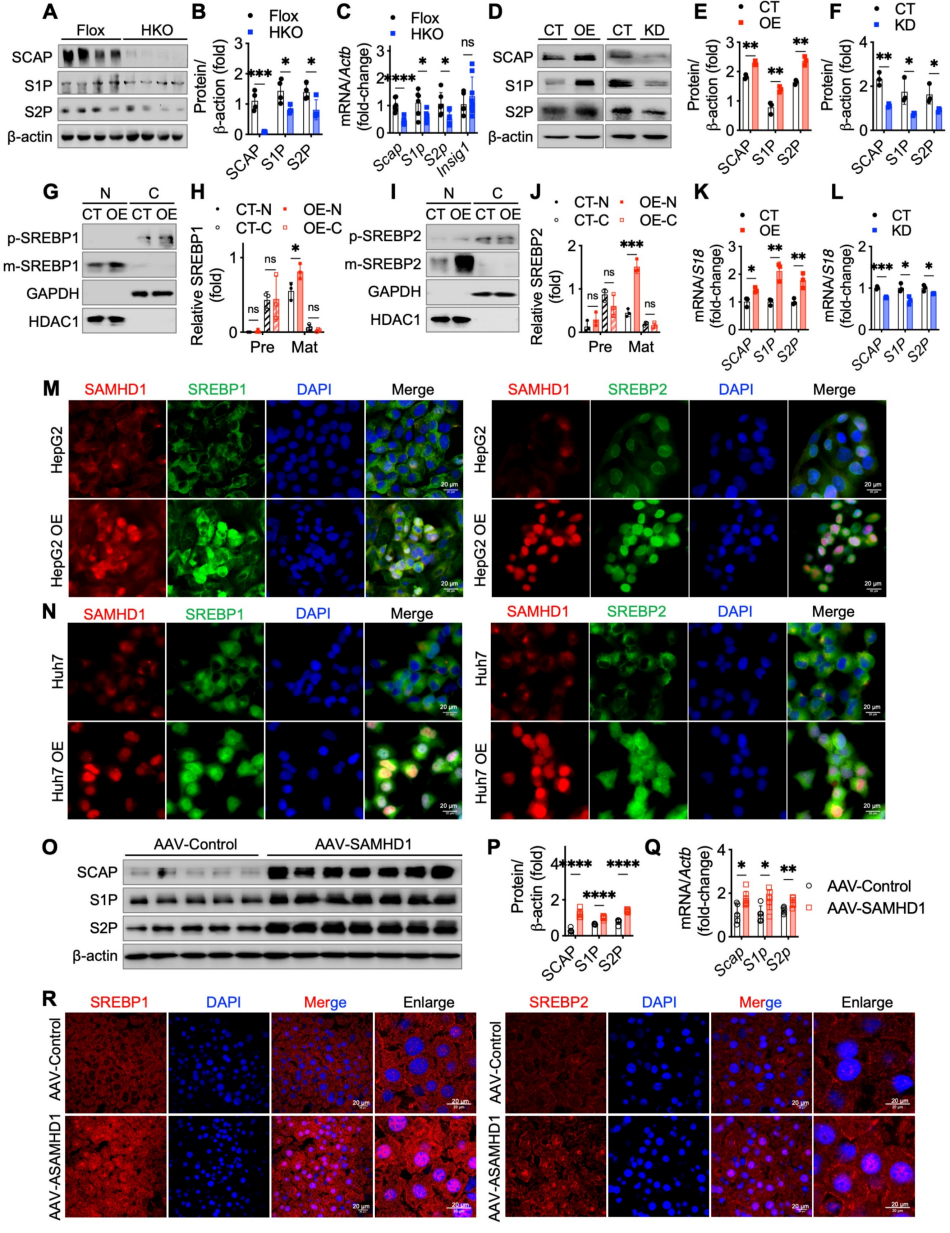
** Hepatocyte SAMHD1 promoted the proteolytic activation of SREBPs and upregulated SCAP, S1P, and S2P expression.** (A, B) Representative western blot and corresponding quantification of SCAP, S1P, and S2P protein levels in primary hepatocytes from Flox and HKO mice (n = 4/group). (C) Relative mRNA expression levels of SCAP, S1P, and S2P in primary hepatocytes from Flox (n = 6/group) and HKO mice (n = 8/group). (D-F) Representative western blot and corresponding quantification of SCAP, S1P, and S2P protein levels in SAMHD1-overexpressing (SAMHD1-OE) and control (CT) HepG2 cells, as well as in SAMHD1-knockdown (SAMHD1-KD) and CT HepG2 cells (n = 3/group). (G, H) Representative western blot and corresponding quantification of p-SREBP1 and m-SREBP1 in nuclear and cytosolic fractions of CT and SAMHD1-OE HepG2 cells, with quantification normalized to HDAC1 for the nuclear fraction (N) and GAPDH for the cytosolic fraction (C) (n = 3/group). (I, J) Representative western blot and corresponding quantification of p-SREBP2 and m-SREBP2 in nuclear and cytosolic fractions of CT and SAMHD1-OE HepG2 cells (n = 3/group). (K, L) Relative mRNA expression levels of SCAP, S1P, and S2P in SAMHD1-OE and CT HepG2 cells, as well as in SAMHD1-KD and CT HepG2 cells (n = 3/group). (M, N) Representative immunofluorescence images showing SAMHD1 and SREBP1 or SREBP2 staining in SAMHD1-OE HepG2 or Huh7 cells and CT cells. Scale bars: 20 µm. (O, P) Representative western blot and corresponding quantification of SCAP, S1P, and S2P protein levels in liver lysates from AAV-Control and AAV-SAMHD1 mice. (Q) Relative mRNA levels of SCAP, S1P, and S2P in liver lysates from the indicated groups. (R) Representative immunofluorescence images showing SREBP1 and SREBP2 in liver sections from the indicated groups. Scale bars: 20 µm. Data are presented as means ± SD. p < 0.05 (*), p < 0.01 (**), p < 0.001 (***), and p < 0.0001 (****). Two-tailed Student's t-test was used for two-group comparisons in (B), (C), (E), (F), (H), (J-L), (P) and (Q).

We then examined the role of SAMHD1 in SREBP proteolytic cleavage by analyzing key processing factors, including SREBP cleavage-activating protein (SCAP), Site-1 protease (S1P), and Site-2 protease (S2P). *In vitro*, SAMHD1 knockout in primary mouse hepatocytes markedly reduced SCAP, S1P, and S2P protein levels (Fig. 7A, B), and qPCR analysis showed decreased mRNA levels of these factors, without affecting Insulin-induced gene 1 (Insig1), a negative regulator of SREBP cleavage (Fig. 7C). Conversely, SAMHD1 overexpression in HepG2 cells increased SCAP, S1P, and S2P expression, whereas SAMHD1 knockdown reduced their levels (Fig. 7D-F, K, L). Subcellular fractionation assays confirmed that SAMHD1 promotes SREBP cleavage, as indicated by increased nuclear accumulation of active SREBP1 and SREBP2 *in vitro* (Fig. 7G-J). Confocal microscopy further revealed enhanced nuclear localization of SREBP1 and SREBP2 in SAMHD1-overexpressing HepG2 and Huh7 cells (Fig. 7M, N). These findings were recapitulated *in vivo* using AAV-mediated, hepatocyte-specific overexpression of SAMHD1, which enhanced nuclear localization of SREBP1 and SREBP2 (Fig. 7R) and increased SCAP, S1P, and S2P expression at both protein and mRNA levels (Fig. 7O-Q). Together, these results indicate that SAMHD1 upregulates key regulators of SREBP proteolytic processing, thereby promoting SREBP activation and hepatic lipid accumulation.

**Figure 8:**
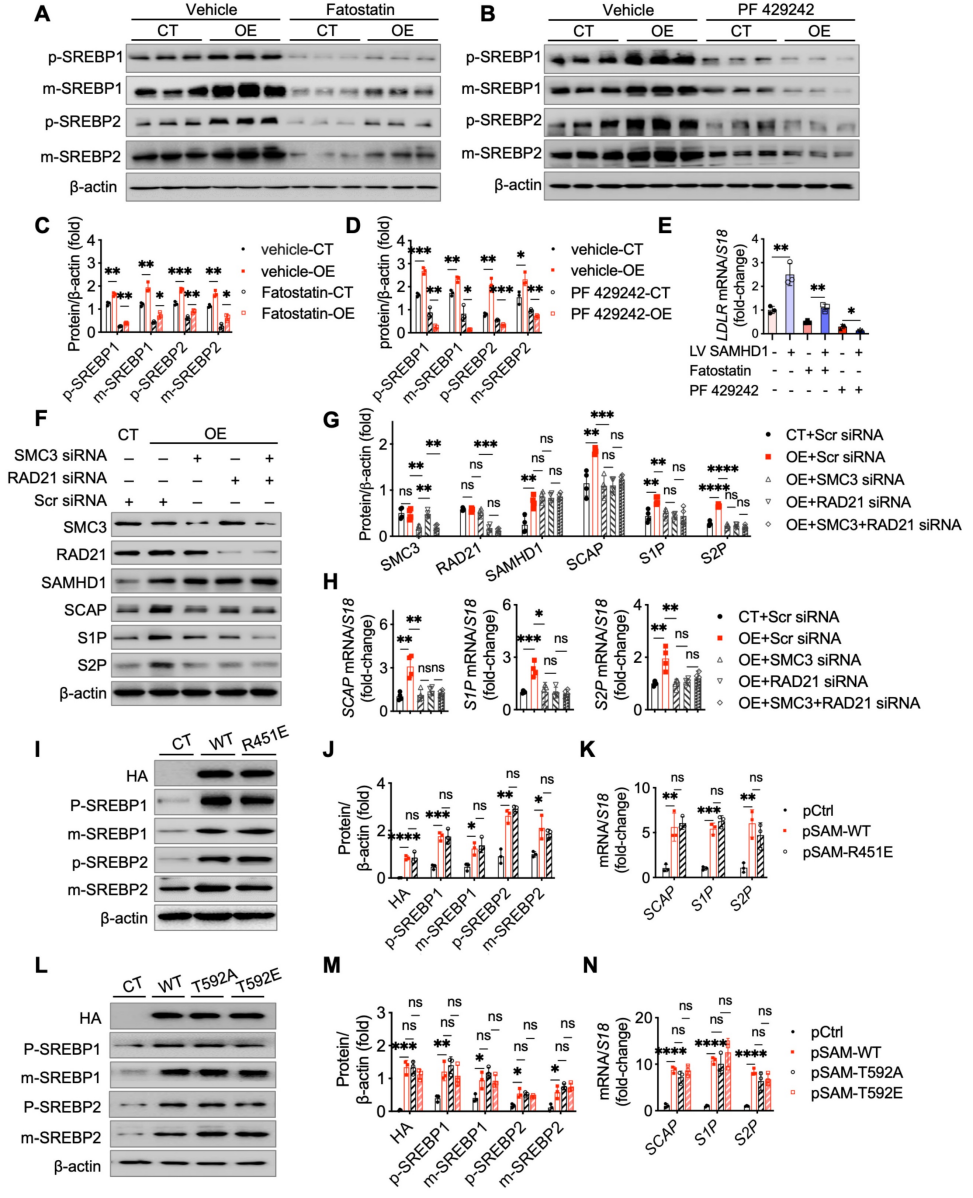
** SAMHD1 promoted SREBP1 and SREBP2 activation by enhancing their proteolytic process in a cohesin complex-dependent manner.** (A, C) Representative western blot and corresponding quantification of precursor and mature forms of SREBP1 and SREBP2 in SAMHD1-overexpressing (SAMHD1-OE) and control (CT) HepG2 cells treated with fatostatin (10 µM, 24 h) or vehicle control, normalized to β-actin (n = 3/group). (B, D) Representative western blot and corresponding quantification of precursor and mature forms of SREBP1 and SREBP2 in SAMHD1-OE and CT HepG2 cells treated with FP 429242 (10 µM, 24 h) or vehicle control, normalized to β-actin (n = 3/group). (E) Relative mRNA expression levels of LDLR in SAMHD1-OE and CT HepG2 cells after treatment with fatostatin, FP 429242, or vehicle control (n = 3/group). (F-H) Representative western blot and corresponding quantification of indicated protein levels, and relative mRNA expression levels of SCAP, S1P, and S2P in SAMHD1-OE and CT HepG2 cells following transfection with scrambled siRNA, RAD21 siRNA, SMC3 siRNA, or a mixture of RAD21 and SMC3 siRNAs (n = 4/group). (I, J) Representative western blot and corresponding quantification of HA, SREBP precursor, and mature forms in HepG2 cells transfected with expression vectors for wild-type SAMHD1 (SAMHD1 WT) or R451E mutant, normalized to β-actin (n = 3/group). (K) Relative mRNA levels of SCAP, S1P, and S2P in HepG2 cells transfected with expression vectors for SAMHD1 WT or R451E mutant (n = 3/group). (L, M) Representative western blot and corresponding quantification of HA, SREBP precursor, and mature forms in HepG2 cells transfected with expression vectors for SAMHD1 WT, T592A, or T592E mutants, normalized to β-actin (n = 3/group). (N) Relative mRNA levels of SCAP, S1P, and S2P in HepG2 cells transfected with expression vectors for SAMHD1 WT, T592A, or T592E mutants (n = 3/group). Data are presented as means ± SD. p < 0.05 (*), p < 0.01 (**), p < 0.001 (***), and p < 0.0001 (****). Two-tailed Student's t-test was used for two-group comparisons in (C-E), (G), (H), (J), (K), (M), and (N).

To investigate whether SAMHD1 facilitates SREBP cleavage through SCAP-mediated ER-to-Golgi migration and proteolytic processing in the Golgi, two inhibitors were used: fatostatin to block SCAP-SREBP migration and PF-429242, a selective S1P inhibitor. Fatostatin treatment reduced mature SREBP levels in SAMHD1-overexpressing HepG2 cells, but levels remained higher than in fatostatin-treated control HepG2 cells, indicating partial inhibition of SAMHD1-induced SREBP proteolytic activation (Fig. 8A, C). In contrast, PF-429242 treatment completely abolished the elevated mature SREBP levels in SAMHD1-overexpressing cells (Fig. 8B, D). qPCR analysis showed that fatostatin partially inhibited LDLR mRNA expression in SAMHD1-overexpressing cells compared to control HepG2 cells, whereas PF-429242 treatment abolished the SAMHD1-induced increase in LDLR mRNA (Fig. 8E). These findings suggest that SAMHD1 enhances SREBP activation by promoting SCAP-mediated ER-to-Golgi migration and subsequent proteolytic cleavage by S1P and S2P in the Golgi.

Our previous study demonstrated that SAMHD1 interacts with cohesin complex 18, which regulates gene transcription through its effects on genome organization and chromatin structure 24. To investigate whether this interaction influences SREBP processing, we knocked down the core cohesin subunits SMC3 and RAD21 in SAMHD1-overexpressing HepG2 cells. This resulted in reduced expression levels of SCAP, S1P, and S2P to levels observed in control cells, indicating that SAMHD1-mediated upregulation of these proteins depends on the cohesin complex (Fig. 8F-H). Given SAMHD1's role as a dNTPase in regulating cellular dNTP pools, we investigated the impact of its dNTPase activity on SREBP activation. Cells overexpressing either wild-type SAMHD1 or the dNTPase-defective R451E mutant, which disrupts SAMHD1 tetramer formation—a critical step for its dNTPase function 25, 26—showed similar levels of SREBP activation and comparable expression of SCAP, S1P, and S2P (Fig. 8I-K). These results suggest that SAMHD1-mediated SREBP activation is independent of its dNTPase activity. Additionally, phosphorylation of T592 is essential for regulating SAMHD1's dNTPase activity by modulating the dynamics of its catalytically active tetramer 27. Both the T592E phosphomimic and the T592A phospho-ablative mutant impair dNTPase activity 28, 29, yet overexpression of either T592 mutant had no effect on SREBP cleavage compared to wild-type SAMHD1 (Fig. 8L-N). These findings further support that SAMHD1 enhances SREBP activation through SCAP-mediated trafficking and the subsequent proteolytic processing, independent of its dNTPase activity.

## Discussion

SAMHD1, a cellular enzyme involved in nucleotide metabolism and immune regulation, has an unclear role in MASLD. We found that hepatic SAMHD1 expression was elevated in both clinical MASLD samples and diet-induced MASLD mouse models. *In vitro*, fatty acids, cholesterol, and IFN-γ upregulated SAMHD1 expression. Functional assays showed that SAMHD1 overexpression promoted lipid droplet accumulation, while its knockdown reduced accumulation in HepG2 and Huh7 cells. *In vivo*, hepatocyte-specific SAMHD1 overexpression increased liver steatosis, while hepatocyte-specific SAMHD1 knockout alleviated liver steatosis and injury in a GAN diet-induced MASLD model. Mechanistically, SAMHD1 enhances SREBP1 and SREBP2 signaling by promoting their proteolytic activation via upregulation of SCAP, S1P, and S2P expression in a cohesin complex-dependent manner. These findings reveal a previously unrecognized role for SAMHD1 in hepatic lipid metabolism and liver steatosis during MASLD progression.

In this study, we observed elevated hepatic SAMHD1 expression in MASLD patients and in diet-induced MASLD mouse models, in both hepatocytes and macrophages including Kupffer cells. SAMHD1's role in macrophages has been extensively studied, with its loss promoting M1 polarization and activating the NF-κB pathway 30. Therefore, in this study, we focused on hepatocyte SAMHD1. SAMHD1, identified as a homolog of the murine IFN-γ-inducible gene Mg11 31, is induced by IFN-γ in murine macrophages 32, but not in activated CD4⁺ T cells 33, suggesting cell type-specific regulation by IFN-γ 34. Our results showed that IFN-γ treatment upregulated SAMHD1 expression in HepG2 and Huh7 cells, constant with findings in primary human hepatocytes 35. We also observed increased IFN-γ receptor 1 expression and enhanced STAT1 activation in cells cultured in lipid-rich media with palmitic acid, oleic acid or cholesterol, likely contributing to increased SAMHD1 expression. Liver injury and inflammation further elevate IFN-γ receptor expression on hepatocyte membranes, amplifying their responsiveness to IFN-γ 36. Moreover, IFN-γ levels are elevated in MASH patients and positively correlate with disease severity 37. Taken together, dyslipidemia- and inflammation-induced upregulation of IFN-γ receptor, along with elevated IFN-γ in MASLD, likely drive SAMHD1 upregulation in hepatocytes.

Our study showed that hepatocyte SAMHD1 promotes lipid accumulation. Overexpression of SAMHD1 in HepG2 and Huh7 cells increased intracellular lipid droplets, and elevated TG and TC levels, especially under palmitic and oleic acid treatment. Conversely, SAMHD1 knockdown reduced lipid accumulation. *In vivo*, hepatocyte-specific SAMHD1 overexpression resulted in significant hepatic lipid accumulation, increasing serum and hepatic TG, TC, and LDL-C levels. These findings align with previous studies showing that mutations in SAMHD1 disrupt cholesterol biosynthesis in a zebrafish model of type I interferonopathies 38. Our data also show that SAMHD1 promotes steatosis in MASLD, with hepatic SAMHD1 expression correlating with steatosis severity in diet-induced MASLD mouse models. Hepatocyte-specific SAMHD1 knockout mice had reduced hepatic lipid accumulation, apoptosis, and ER stress under GAN diet conditions, suggesting a protective role of SAMHD1 deficiency in MASLD progression.

Our study demonstrated that SAMHD1 enhances SREBP1 and SREBP2 activation by promoting their proteolytic processing. Although RNA sequencing of primary hepatocytes from HKO mice showed downregulation of *Srebf1*, luciferase and CHX chase assays indicated that SAMHD1 does not directly affect SREBP transcription or protein stability. This prompted us to explore SAMHD1's effect on SREBP maturation. SREBPs are critical transcription factors regulating lipogenesis, with SREBP-1 primarily involved in fatty acid and triacylglycerol synthesis and SREBP-2 in cholesterol biosynthesis 39. Synthesized as ER-bound precursors, SREBPs are escorted by SCAP to the Golgi, where sequential cleavage by S1P and S2P releases the N-terminal domain (nSREBP), which then translocate to nucleus to activate lipogenic gene expression 40-42. In our study, confocal imaging and cellular fractionation assays showed increased nuclear localization of SREBPs in SAMHD1-overexpressing HepG2 cells and liver sections from hepatocyte SAMHD1-overexpressing mice. Mechanistically, SAMHD1 enhances SREBP activation by upregulating SCAP, S1P, and S2P—key regulators of SREBP maturation. Pharmacological inhibitor assays further supported this mechanism: Fatostatin, which blocks SCAP-mediated ER-to-Golgi transport, partially attenuated SAMHD1-induced SREBP activation, while PF-429242, an S1P inhibitor, completely abolished it, indicating a multi-target regulatory process involving both SCAP-mediated transport and subsequent S1P-dependent cleavage. Notably, SREBPs regulate their own expression in a feedback loop via sterol response elements (SREs) in their promoters 43, which likely explains the observed changes in SREBP mRNA levels following SAMHD1 modulation. Our group previously demonstrated that SAMHD1 interacts with the cohesin complex 18, and here we demonstrated that SAMHD1 upregulates SCAP, S1P, and S2P in a cohesin complex-dependent manner. The cohesin complex regulates gene transcription by organizing chromatin loops to bring enhancers and promoters into proximity, interacting with transcription factors, and shaping the epigenetic landscape 44, 45. Knockdown of cohesin components SMC3 and RAD21 significantly reduced SAMHD1-induced upregulation of SCAP, S1P, and S2P. These findings align with studies showing that cohesin complex regulates lipid metabolism, as evidenced by the downregulation of lipid metabolism genes following the deletion of cohesin subunit SA1 46, and the promotion of cohesin-mediated enhancer-promoter interactions at the Insig2 locus by methyltransferase SETDB2 methyltransferase, which inhibits SREBP maturation 47. Together, these data support a model in which SAMHD1 modulates lipid metabolism through cohesin complex-dependent regulation of SREBP processing.

SREBP-mediated lipogenesis plays a critical role in hepatic lipid metabolism, with its hyperactivation implicated in the progression of MASLD 48. However, complete inhibition of SREBP has proven deleterious. For instance, liver-specific deletion of SCAP reduces hepatic steatosis but exacerbates liver injury, fibrosis, and carcinogenesis due to impaired lipid homeostasis and increased inflammation 49. These findings highlight the need for nuanced regulation rather than complete inhibition of SREBP signaling. Our study suggests that SAMHD1, which modulates SREBP activity, could serve as a novel therapeutic target to fine-tune this pathway in MASLD. Targeting SAMHD1, for instance with siRNA encapsulated in hepatocyte-targeted lipid nanoparticles 50, may allow controlled modulation of SREBP-mediated lipogenesis, reducing lipid accumulation without the deleterious effects seen with broad SREBP inhibition. This approach could offer a safer and more precise strategy to mitigate disease progression in MASLD. To further elucidate the mechanistic role of SAMHD1, future studies utilizing chromosome conformation capture (3C) and related techniques will be essential to determine whether SAMHD1 promotes long-range chromatin interactions through cohesin complex, thus driving gene expression programs that regulate lipid metabolism.

In conclusion, hepatic SAMHD1 expression is elevated in MASLD and is closely correlates with the severity of steatosis. Induced by lipids and IFN-γ, SAMHD1 promotes lipid droplet accumulation, worsening liver steatosis and injury in diet-induced MASLD models through activation of the SREBP1 and SREBP2 pathways. Mechanistically, SAMHD1 enhances SREBP processing by upregulating SCAP, S1P, and S2P in a cohesin complex-dependent manner, positioning it as a promising target for modulating hepatic lipid metabolism.

## Supplementary Material

Supplementary figure, tables, and information.
