# Supplementary material for: Hepatocyte SAMHD1 Deficiency Attenuates Hepatic Steatosis via Suppression of SREBP Activation in a Mouse Model of Metabolic-Associated Steatotic Liver Disease
Source: Int J Biol Sci. 2026 Jan 1;22(2):876–94. doi: 10.7150/ijbs.125688 (PMC12781173; doi:10.7150/ijbs.125688)
Supplement: Supplementary file 1 — Supplementary figure, tables, and information. [file ijbsv22p0876s1.pdf]

Supplementary materials

Patient ID	Age (yrs)	Sex	Liver Steatosis (%)	Lobular inflammation under 200X	Hepatocellular ballooning	NAFLD Activity Score
1	59	F	33-66	≥4 foci	Moderate	6
2	28	F	33-66	≥4 foci	Moderate	6
3	57	F	33-66	2-4 foci	Moderate	5
4	53	F	5-33	2-4 foci	Moderate	4
5	60	M	5-33	2-4 foci	Moderate	4
6	79	M	33-66	2-4 foci	Moderate	5
7	50	F	<5	No	No	0
8	50	F	<5	No	No	0
9	69	F	<5	No	No	0
10	48	M	<5	No	No	0
11	73	M	<5	No	No	0
12	51	F	<5	No	No	0
13	74	F	<5	No	No	0

Supplementary Table 1. Baseline Characteristics of Study Participants (Related to Figure 1). The NAFLD Activity Score (NAS) was calculated based on histological evaluation of liver biopsy samples, assessing the degree of steatosis, lobular inflammation, and hepatocellular ballooning, as described by Kleiner et al [1]. The NAS provides a composite score that ranges from 0 to 8, with higher scores correlating with more severe disease activity in NAFLD.

Target	Species	Application	Sequence (5'-3')
SAMHD1	Human	qPCR	Forward primer: AGGACCACTTGAATCACCTGT<br>Reverse primer: GCTTTTGTTTTCAGGACGCC
SREBP1	Human	qPCR	Forward primer: CACCGTTTCTTCGTGGATGG<br>Reverse primer: TCCCGGAATAGCTGAGTCAC
SREBP2	Human	qPCR	Forward primer: CTCCATTGACTCTGAGCCAGGA<br>Reverse primer: GAATCCGTGAGCGGTCTACCAT
ACC1	Human	qPCR	Forward primer: TTCACTCCACCTTGTCAGCGGA<br>Reverse primer: GTCAGAGAAGCAGCCCATCACT
FASN	Human	qPCR	Forward primer: TTTTGCTTCACTCCCCACAC<br>Reverse primer: CCACCATCTTCAGCCCCTG
LDLR	Human	qPCR	Forward primer: TGTCTCTGTTGCGGATACCA<br>Reverse primer: TCTTGATCTTGGCGGGAGTT
SCAP	Human	qPCR	Forward primer: TCCTCATCGGCTACTTCACC<br>Reverse primer: TGGACAGGACAGTGGTGAAA
S1P	Human	qPCR	Forward primer: GCATGGGAGTCACAGGAAGA<br>Reverse primer: AAGGCCATAAGACCGAGGAG
S2P	Human	qPCR	Forward primer: CTTTTACAGTTGGGGACGGC<br>Reverse primer: GTCAGCCATCATTTGTGCCA
IFNGR1	Human	qPCR	Forward primer: AGTGCTTAGCCTGGTATTCATCTG<br>Reverse primer: GGCTGGTATGACGTGATGAGTG
IFNGR2	Human	qPCR	Forward primer: GGCTCCCTCATCATCAGGTT<br>Reverse primer: CAATGAAATGGAGTTGCTTCTGA
RPS18	Human	qPCR	Forward primer: GCAGAATCCACGCCAGTACAAG<br>Reverse primer: GCTTGTTGTCCAGACCATTGGC
SAMHD1	Mouse	qPCR	Forward primer: CCGAGAGAAGAAAGTGCTGG<br>Reverse primer: ACTCCCAGATCTTCCAGACG

SREBP1	Mouse	qPCR	Forward primer: AGGTCACCGTTTCTTTGTGG<br>Reverse primer: AGAACTCCCTGTCTCCGTCA
SREBP2	Mouse	qPCR	Forward primer: AGAAAGAGCGGTGGAGTCCTTG<br>Reverse primer: GAACTGCTGGAGAATGGTGAGG
ACC1	Mouse	qPCR	Forward primer: CTGGCTGCATCCATTATGTCA<br>Reverse primer: TGGTAGACTGCCCGTGTGAA
FASN	Mouse	qPCR	Forward primer: CTGCCACAACTCTGAGGACA<br>Reverse primer: CGGATCACCTTCTTGAGAGC
LDLR	Mouse	qPCR	Forward primer: TGACTCAGACGAACAAGGCT<br>Reverse primer: TCGGTCTCCATCACACACAA
SCAP	Mouse	qPCR	Forward primer: CCACACACCATCACATTGCA<br>Reverse primer: GATGCCAATCCAGACAACGG
S1P	Mouse	qPCR	Forward primer: CTGGTCAGGTTACCTTGCCATC<br>Reverse primer: GCTTCACGGTGGAAGTGTGCTC
S2P	Mouse	qPCR	Forward primer: ATCACCAGTCCAGCAGCTAAGG<br>Reverse primer: AGGCAAGAGGATTACTGGGAGG
β-actin	Mouse	qPCR	Forward primer: GGCTGTATTCCCCTCCATCG<br>Reverse primer: CCAGTTGGTAACAATGCCATGT
SAMHD1	Human	siRNA	Sense: GAUUCAUUGUGGCCAUAUATT<br>Anti-sense: UAUAUGGCCACAAUGAAUCTT
SMC3	Human	siRNA	Sense: CAGCGGUUGGCUUUAUUGC<br>Anti-sense: GCAAUAAAGCCAACCGCUG
RAD21	Human	siRNA	Sense: CUCCAAAUAUCUGUCAGCUAA<br>Anti-sense: UUAGCUGACAGAUAUUUGGAG

Supplementary Table 2. Sequence of primers and siRNA used in this manuscript.

Antibody	CatLog #	Source	Application	Dilution
ACC1	21923-1-AP	Proteintech	WB	1:4000
BiP	CST3177	CST	WB	1:1000
CD68	28058-1-AP	Proteintech	IHC	1:1000
CHOP	CST2895	CST	WB	1:1000
F4/80	28463-1-AP	Proteintech	IF	1:250
F4/80	ab300421	Abcam	IF	1:250
F4/80	GB11027	Servicebio	IHC	1:1000
FASN	sc-48357	Santa Cruz	WB	1:200
Flag	F1804	Sigma-Aldrich	WB	1:4000
GAPDH	T0004	Affinity	WB	1:4000
HA	51064-2-AP	Proteintech	WB	1:5000
HDAC1	sc-81598	Santa Cruz	WB	1:200
HNF4α	ab199431	Abcam	IF	1:250
IFNGR1	10808-1-AP	Proteintech	WB	1:1000
LDLR	10785-1-AP	Proteintech	WB	1:2000
Phospho-STAT1 (Tyr701)	CST7649	CST	WB	1:1000
RAD21	ab217678	Abcam	WB	1:1000
S1P	DF4159	Affinity	WB	1:1000
S2P	sc-293341	Santa Cruz	WB	1:200
SAMHD1	12586-1-AP	Proteintech	WB/IP/IHC	1:2000/1:200/1:400
SAMHD1	TA502024S	Origene	WB/IF	1:1000/1:50
SCAP	ab308060	Abcam	WB	1:1000
SMC3	ab128919	Abcam	WB	1:1000
SREBP1	sc-13551	Santa Cruz	WB	1:200
SREBP1	14088-1-AP	Proteintech	IF	1:50
SREBP2	28212-1-AP	Proteintech	WB/IF	1:2000/1:50
STAT1	ab92506	Abcam	WB	1:2000
β-Actin	AF7018	Affinity	WB	1:5000
Goat Anti-Rabbit IgG (HRP conjugate)	E-AB-1003	Elabscience	WB	1:4000

Goat Anti-Mouse IgG (HRP conjugate)	E-AB-1001	Elabscience	WB	1:4000
Goat anti-Mouse IgG (Alexa Fluor 555 conjugate)	4409	CST	IF	1:100
Goat anti-Rabbit IgG (Alexa Fluor 488 conjugate)	4412	CST	IF	1:200
Goat anti- Rabbit IgG (Alexa Fluor 555 conjugate)	4413	CST	IF	1:100
Goat anti- Mouse IgG (Alexa Fluor 488 conjugate)	4408	CST	IF	1:200

Supplementary Table 3. Information regarding antibodies used in this manuscript.

1 Kleiner DE, Brunt EM, Van Natta M, Behling C, Contos MJ, Cummings OW, et al. Design and validation of a histological scoring system for nonalcoholic fatty liver disease. Hepatology 2005; 41: 1313-21.

Supplementary Figure

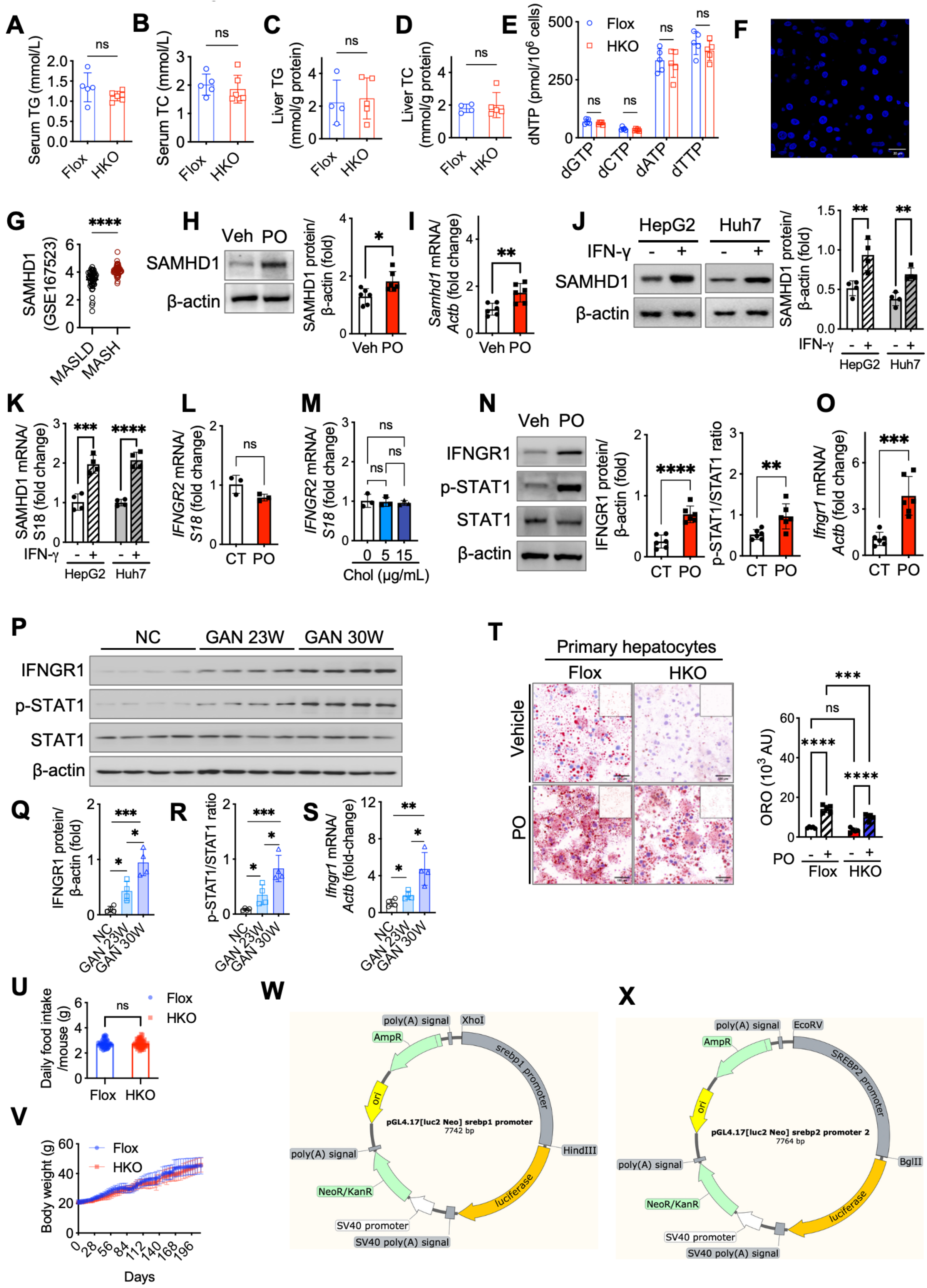

Supplementary Figure 1. (A, B) Serum TG and TC levels in Flox (n = 5) and HKO mice (n = 6) aged 6–8 weeks on normal chow. (C, D) Liver TG and TC levels in Flox (n = 4) and HKO mice (n = 5) aged 6–8 weeks on normal chow. (E) dNTP levels in primary hepatocytes from Flox and HKO mice (n = 5/group). (F) Negative control staining for Figure 1B and C using isotype control antibodies. (G) Scatter plots showing SAMHD1 expression levels in the GEO dataset GSE167523. The y-axis represents log2-transformed TPM-normalized expression values of SAMHD1. The dataset includes liver transcriptomic data from MASLD patients with steatosis alone (n = 51) and MASH, the progressive steatohepatitis form (n = 47). (H, I) Western blot analysis and quantification of SAMHD1 protein and relative SAMHD1 mRNA levels in primary mouse hepatocytes cultured in control medium or supplemented with PA/OA (n = 6/group). (J) Western blot analysis and quantification of SAMHD1 in HepG2 and Huh7 cells treated with 20 ng/mL IFN-γ for 24 hours (n = 4/group). (K) SAMHD1 mRNA expression after IFN-γ treatment (n = 4/group). (L) Relative IFNGR2 mRNA expression in HepG2 cells cultured in control medium or supplemented with PA/OA (n = 3/group). (M) Relative IFNGR2 mRNA expression in HepG2 cells treated with cholesterol (n = 3/group). (N) Western blot analysis and quantification of IFNGR1 and the relative phosphorylated-STAT1 ratio in primary mouse hepatocytes cultured in control medium or supplemented with PA/OA (n = 6/group). (O) IFNGR1 mRNA expression in primary mouse hepatocytes after PA/OA treatment. (P, Q, R) Western blot analysis and quantification of IFNGR1 and the relative phosphorylated-STAT1 ratio in liver samples from mice fed a normal chow (NC) diet or a GAN diet for 23 or 30 weeks (n = 4/group). (S) Relative IFNGR1 mRNA levels in liver samples from the indicated groups. (T) Representative ORO staining images of primary hepatocytes from Flox or HKO mice, with or without PA/OA treatment. Scale bars: 100 μm. Quantification of ORO-stained areas using ImageJ software (n = 5/group). (U) Daily food intake of Flox and HKO mice on a GAN diet. (V) Body weight growth curves of Flox and HKO mice on a GAN diet. (W, X)

Schematic diagrams of pGL4.17[luc2/Neo]-SREBP1 promoter plasmid and pGL4.17[luc2/Neo]-SREBP2 promoter plasmid. Data are presented as mean ± SD. Statistical significance is indicated as follows: *$p < 0.05$, **$p < 0.01$, ***$p < 0.001$, and ****$p < 0.0001$. Two-tailed Student's t-test was used for two-group comparisons in (A–E), (G–O) and (Q–V).

Supplementary Information

Plasmids for SAMHD1 mutants (R451E, T592A, and T592E) were constructed using the pLVX-HA-SAMHD1-IRES-Puro plasmid. Linear DNA sequences encoding SAMHD1 from lysine 332 to the C-terminus were synthesized and inserted between the AfeI and BamHI restriction sites by Sangon Biotech (Shanghai, China).The AfeI and BamHI restriction sites are highlighted with underscores, and the corresponding mutated amino acids are shown in bold font, as detailed below.

SAMHD1 R451E:
AGCGCTTTATTAAGTTTGCCCGTGTCTGTGAAGTAGACAATGAGTTGCGTATTTG
TGCTAGAGATAAGGAAGTTGGAAATCTGTATGACATGTTCCACACTCGCAACTCT
TTACACCGTAGAGCTTATCAACACAAAGTTGGCAACATTATTGATACAATGATTA
CAGATGCTTTCCTCAAAGCAGATGACTACATAGAGATTACAGGTGCTGGAGGAA
AAAAGTATCGCATTTCTACAGCAATTGACGACATGGAAGCCTATACTAAGCTGA
CAGATAACATTTTTCTGGAGATTTTATACTCTACTGATCCCAAATTGAAAGACGC
ACGAGAGATTTTAAAACAAATTGAATAC**GAA**AATCTATTCAAGTATGTGGGTGA
GACGCAGCCAACAGGACAAATAAAGATTAAAAGGGAGGACTATGAATCTCTTCC
AAAAGAGGTTGCCAGTGCTAAACCCAAAGTATTGCTAGACGTGAAACTGAAGGC
TGAAGATTTTATAGTGGATGTTATCAACATGGATTATGGAATGCAAGAAAAGAA
TCCAATTGATCATGTTAGCTTCTATTGTAAGACTGCCCCCAACAGAGCAATCAGG
ATTACTAAAAACCAGGTTTCACAACTTCTGCCAGAGAAATTTGCAGAGCAGCTG
ATTCGAGTATATTGTAAGAAGGTGGACAGAAAGAGTTTGTATGCCGCAAGACAA
TATTTTGTTCAGTGGTGTGCAGACAGAAATTTCACCAAGCCGCAGGATGGCGATG
TTATAGCCCCACTCATAACACCTCAAAAAAAGGAATGGAACGACAGTACTTCAG
TCCAAAATCCAACTCGCCTCCGAGAAGCATCCAAAAGCAGAGTCCAGCTTTTTAA
AGATGACCCAATGTGAGGATCC

SAMHD1 T592A:
AGCGCTTTATTAAGTTTGCCCGTGTCTGTGAAGTAGACAATGAGTTGCGTATTTG
TGCTAGAGATAAGGAAGTTGGAAATCTGTATGACATGTTCCACACTCGCAACTCT
TTACACCGTAGAGCTTATCAACACAAAGTTGGCAACATTATTGATACAATGATTA
CAGATGCTTTCCTCAAAGCAGATGACTACATAGAGATTACAGGTGCTGGAGGAA
AAAAGTATCGCATTTCTACAGCAATTGACGACATGGAAGCCTATACTAAGCTGA
CAGATAACATTTTTCTGGAGATTTTATACTCTACTGATCCCAAATTGAAAGACGC
ACGAGAGATTTTAAAACAAATTGAATACCGTAATCTATTCAAGTATGTGGGTGAG
ACGCAGCCAACAGGACAAATAAAGATTAAAAGGGAGGACTATGAATCTCTTCCA
AAAGAGGTTGCCAGTGCTAAACCCAAAGTATTGCTAGACGTGAAACTGAAGGCT
GAAGATTTTATAGTGGATGTTATCAACATGGATTATGGAATGCAAGAAAAGAAT
CCAATTGATCATGTTAGCTTCTATTGTAAGACTGCCCCCAACAGAGCAATCAGGA
TTACTAAAAACCAGGTTTCACAACTTCTGCCAGAGAAATTTGCAGAGCAGCTGAT
TCGAGTATATTGTAAGAAGGTGGACAGAAAGAGTTTGTATGCCGCAAGACAATA
TTTTGTTCAGTGGTGTGCAGACAGAAATTTCACCAAGCCGCAGGATGGCGATGTT
ATAGCCCCACTCATA**GCA**CCTCAAAAAAAGGAATGGAACGACAGTACTTCAGTC

CAAAATCCAACTCGCCTCCGAGAAGCATCCAAAAGCAGAGTCCAGCTTTTTAAA
GATGACCCAATGTGAGGATCC

SAMHD1 T592E:
AGCGCTTTATTAAGTTTGCCCGTGTCTGTGAAGTAGACAATGAGTTGCGTATTTG
TGCTAGAGATAAGGAAGTTGGAAATCTGTATGACATGTTCCACACTCGCAACTCT
TTACACCGTAGAGCTTATCAACACAAAGTTGGCAACATTATTGATACAATGATTA
CAGATGCTTTCCTCAAAGCAGATGACTACATAGAGATTACAGGTGCTGGAGGAA
AAAAGTATCGCATTTCTACAGCAATTGACGACATGGAAGCCTATACTAAGCTGA
CAGATAACATTTTTCTGGAGATTTTATACTCTACTGATCCCAAATTGAAAGACGC
ACGAGAGATTTTAAAACAAATTGAATACCGTAATCTATTCAAGTATGTGGGTGAG
ACGCAGCCAACAGGACAAATAAAGATTAAAAGGGAGGACTATGAATCTCTTCCA
AAAGAGGTTGCCAGTGCTAAACCCAAAGTATTGCTAGACGTGAAACTGAAGGCT
GAAGATTTTATAGTGGATGTTATCAACATGGATTATGGAATGCAAGAAAAGAAT
CCAATTGATCATGTTAGCTTCTATTGTAAGACTGCCCCCAACAGAGCAATCAGGA
TTACTAAAAACCAGGTTTCACAACTTCTGCCAGAGAAATTTGCAGAGCAGCTGAT
TCGAGTATATTGTAAGAAGGTGGACAGAAAGAGTTTGTATGCCGCAAGACAATA
TTTTGTTCAGTGGTGTGCAGACAGAAATTTCACCAAGCCGCAGGATGGCGATGTT
ATAGCCCCACTCATA**GAA**CCTCAAAAAAAGGAATGGAACGACAGTACTTCAGTC
CAAAATCCAACTCGCCTCCGAGAAGCATCCAAAAGCAGAGTCCAGCTTTTTAAA
GATGACCCAATGTGAGGATCC
